# Breast milk lacking anti-human immunodeficiency virus activity promotes exocytosis of HIV from the mother’s mammary epithelium and transcytosis of virus via the infant’s tonsil and intestinal epithelial cells

**DOI:** 10.1016/j.virol.2025.110689

**Published:** 2025-09-11

**Authors:** Nicole T. Padilla, Xiaodan Cai, Rossana Herrera, Kristina Rosbe, Sharof M. Tugizov

**Affiliations:** a Department of Medicine, University of California, San Francisco, CA, USA; b Department of Otolaryngology, University of California, San Francisco, CA, USA

**Keywords:** Human immunodeficiency virus, Mammary epithelial cells, Breast milk, Endocytosis, Transcytosis, Exocytosis, Protein kinase C, Synaptotagmin 7, HIV mother-to-child transmission

## Abstract

Although mammary epithelial cells (MECs) and breast milk facilitate mother-to-child transmission (MTCT) of human immunodeficiency virus (HIV-1), the mechanisms underlying these observations remain poorly understood. In this work, we explored the molecular mechanisms associated with HIV-1 transcytosis through MECs and the role of breast milk in promoting viral transmigration through infant tonsils and intestinal epithelia. Our findings revealed that transfer of cell-free HIV-1 from the maternal circulation into breast milk is mediated by its basolateral-to-apical transcytosis through polarized MECs that function as a blood-milk barrier. While breast milk samples from 76 % of the HIV-negative donors contained factors that inactivated the virus by disrupting viral envelope glycoprotein gp120, thereby preventing MTCT, the remaining breast milk samples that were incapable of inactivating HIV-1 nonetheless induced viral exocytosis from the apical surface of MECs. Interestingly, these otherwise inactive breast milk samples also promoted a substantial increase in viral internalization, transcytosis, and exocytosis from infant tonsils and gut epithelial cells, ultimately leading to infection of virus-susceptible subepithelial cells. We determined that elevated calcium concentrations in breast milk play an important role in promoting HIV-1 entry, transcytosis, and exocytosis from infant tonsil and gut epithelial cells via activation of protein kinase C and calcium sensor synaptotagmin 7. Collectively, these findings suggest that breast milk samples from different sources may either promote or prevent HIV MTCT by several different mechanisms. Further investigation of this phenomenon may ultimately improve our understanding of the molecular pathogenesis of HIV MTCT and suggest new strategies for its prevention.

## 1. Importance

Although antiretroviral therapy (ART) substantially reduced HIV MTCT, eliminating breastfeeding is a commonly used strategy of HIV-positive mothers seeking to avoid the risk of postnatal HIV transmission to infants. This is unfortunate, as breast milk contains unique factors, including critical innate immune cells, immune cells, proteins, and essential nutrients that promote normal infant development, most notably in resource-limited settings. Although accumulating evidence suggests that breast milk contains factors that inactivate cell-free HIV, our most recent findings revealed that breast milk samples from 24 % of HIV-negative donors cannot inactivate the virus but instead promote increased viral transmigration through the infant’s oral and gut mucosal epithelial cells. Elevated levels of calcium in breast milk and activation of protein kinase C facilitate the efficient release of virus from mammary epithelial cells and its rapid uptake by the infant’s tonsil and gut epithelia. This mechanism may substantially increase HIV MTCT in mothers whose breast milk lacks these antiviral factors. Identification of these factors in breast milk and characterization of one or more of these critical pathways may lead to new strategies for the prevention of HIV MTCT.

## 2. Introduction

Mother-to-child transmission (MTCT) of human immunodeficiency virus-1 (HIV-1) through breastfeeding is an important pathway promoting the spread of the virus (Kuhn et al., 2013; Satomi et al., 2005; Semba et al., 1999). Breast milk of HIV-infected mothers contains cell-free virions as well as HIV-infected CD4^+^ T lymphocytes and macrophages, all of which may initiate the mucosal transmission of the virus (Kuhn et al., 2013; Satomi et al., 2005; Semba et al., 1999). Results of the systematic review revealed viral MTCT via breast milk in ~15–30 % of HIV-1-infected women who were not undergoing treatment with antiretroviral therapy (ART) (Dabis et al., 2004; De Cock et al., 2000; Fowler et al., 2007; Nduati et al., 2001; Powers et al., 2024; White et al., 2014). Moreover, infectious HIV-1 may persist in breast milk despite ART, which significantly reduces MTCT but does not eliminate the virus or its reservoirs (Duong et al., 2022; Goga et al., 2021; Kankasa et al., 2024; Lehman et al., 2008; Low et al., 2015; Ndirangu et al., 2012; Redmond and McNamara, 2015; Ruel et al., 2023; Tassembedo et al., 2024; UNAIDS, 2023; Van de Perre et al., 2021; Van de Perre et al., 2012; Van de Perre et al., 2024).

To initiate systemic HIV-1 infection/acquired immunodeficiency syndrome (AIDS) in a child, the virus must first undergo transmigration across the mammary gland epithelium of the mother and then pass through the epithelial cells lining the oropharyngeal and gastrointestinal mucosa of the child. The molecular mechanisms and the role of breast milk in facilitating this process are poorly understood.

Mammary epithelial cells (MECs) have a polarized organization and serve as critical components of the blood-milk barrier during lactation (Linzell and Peaker, 1971; Pecorini et al., 2009; Truchet et al., 2014; Truchet and Honvo-Houeto, 2017; Truchet and Ollivier-Bousquet, 2009; Wellnitz and Bruckmaier, 2021). The apical membranes of MECs face the lumen, while the basolateral membranes are connected to underlying myoepithelial cells and blood vessels. During lactation, many of the components of breast milk derived from the maternal circulation are transferred via basolateral-to-apical transcytosis through polarized MECs (Monks and Neville, 2004). Detection of cell-free HIV-1 in breast milk (Koulinska et al., 2006; Kuhn et al., 2013; Lewis et al., 1998; Satomi et al., 2005; Semba et al., 1999) suggests that virions may also pass through the MECs via this mechanism. Despite the critical nature of this pathway, the molecular mechanisms leading to transepithelial HIV transport from circulation and into the breast milk are not well-characterized.

Oropharyngeal and gastrointestinal epithelial cells have well-developed tight junctions. These intercellular structures serve as a barrier to paracellular viral transmission and thus prevent the penetration of HIV-1 (Tugizov et al., 2003, 2011, 2012, 2013a, 2013b). HIV-1 transcytosis through mucosal epithelial cells is not highly efficient; only 0.01–0.05 % of virions from an initial inoculum may undergo translocation via this mechanism (Bobardt et al., 2007; Bomsel, 1997; Tugizov et al., 2011; Yasen et al., 2018). More than 90 % of internalized virions remain sequestered in the endosomes (e.g., multivesicular bodies and vacuoles) of epithelial cells (Yasen et al., 2017, 2018). These sequestered virions remain infectious and are released from epithelial cell sequestration in response to inflammatory conditions and interactions with activated peripheral blood mononuclear cells (PBMCs) and CD4^+^ T lymphocytes.

Although breast milk has a virucidal effect on cell-free HIV-1 virions, it does not inhibit cell-associated HIV-1 infection and cell-to-cell viral spread (Lyimo et al., 2009, 2012). Results from earlier studies revealed that breast milk can inhibit HIV infection with cell-free virions in experiments targeting peripheral blood mononuclear cells (PBMCs) *in vitro* and the Bone Marrow-Liver-Thymus humanized mouse model *in vivo* (Lyimo et al., 2009; Wahl et al., 2012, 2015). The molecular mechanisms contributing to the anti-HIV properties of breast milk are also poorly understood.

*Breast milk contains more than 400 proteins, including* cytokines, chemokines, growth factors, hormones, and antimicrobial peptides (Bondt et al., 2021; Christian et al., 2021; Dingess et al., 2017a, 2017b, 2019, 2021; Zhu and Dingess, 2019). Breast milk is also a source of numerous antiviral proteins, including lactoferrin, lysozyme, defensins, and immunoglobulins (Aknouch et al., 2022; Baricelli et al., 2015; Jia et al., 2001; Morniroli et al., 2021; Wedekind and Shenker, 2021).

Macrophages and lymphocytes, including CD4^+^ T cells identified in breast milk, are *in an activated state* and express activation markers, including CD40L, sCD30, and the IL-2 receptor (Field, 2005; Hsu and Nanan, 2018; Ichikawa et al., 2003; Lewis et al., 2017; Tuaillon et al., 2009). Breast milk induces differentiation of monocytes into macrophages (Cai et al., 2024), thereby increasing the susceptibility to HIV-1 infection.

Breast milk also contains approximately threefold higher concentrations of free calcium ions (3–3.5 mM) (Kent et al., 2009; Kulski and Hartmann, 1981; Truchet and Ollivier-Bousquet, 2009) than those found in systemic circulation (1.1–1.3 mM) (Tobias et al., 2023). Increases in calcium signaling via calcium-sensing receptors result in the activation of phosphatidylinositol-kinase and phospholipase C and the accumulation of phosphatidic acid and phosphatidylinositol-(3,4,5)-trisphosphate at the plasma membrane (Canton et al., 2016; Schlam and Canton, 2017). These actions will also lead to the activation of GTPases Rac and Cdc42, which induce actin polymerization and membrane ruffling, triggering macropinocytosis (Bohdanowicz et al., 2013; Canton et al., 2016; Redka et al., 2018; Schlam and Canton, 2017; Veltman et al., 2016; Yamaki et al., 2007).

Calcium influx substantially increases all forms of endocytosis via multiple mechanisms (Wu et al., 2009), including massive membrane invagination (Lariccia et al., 2011) which is critical for both clathrin- and caveolin-mediated endocytosis. Calcium influx promotes critical reorganization of actin filaments and the actin cytoskeleton. The filaments are then cross-linked by the GTPase, dynamin 2, leading to membrane invagination and endocytosis (Galletta et al., 2010; Mooren et al., 2009, 2012; Mooren and Schafer, 2009).

Calcium influx is also critical for exocytosis. Calcium activates protein kinase C (PKC), resulting in the release of intracellular cargo proteins via the secretory pathway into the extracellular environment (Koh et al., 2000; Trexler and Taraska, 2017). Synaptotagmin (SYT) family proteins trigger exocytosis in neurons, epithelial cells, fibroblasts, and macrophages (Hikima et al., 2022; MacDougall et al., 2018; Truchet and Ollivier-Bousquet, 2009) by binding to calcium and inducing the fusion of cargo-containing vesicles with the plasma membrane (Bakhti et al., 2022; Tarquis-Medina et al., 2021).

Calcium, as a secondary messenger, also plays an important role in nearly all steps of the HIV-1 life cycle, including attachment, entry/endocytosis, uncoating, gene expression, assembly, and release of progeny (Chen et al., 2019; Datta et al., 2019; Jha et al., 2011; Kishor et al., 2022; Klug et al., 2022; Miyauchi et al., 2009; Nickoloff-Bybel et al., 2019; Qu et al., 2022). Elevated levels of intracellular calcium also induce calcium-dependent enzymatic processes and the activation of calcium-sensitive transcription factors that promote viral replication and the spread of infection (Bergqvist and Rice, 2001; Bergqvist et al., 2003; Qu et al., 2022).

Here, we investigated the mechanism of HIV-1 transmigration across the mammary epithelium, as well as the contributions of breast milk to HIV-1 transcytosis through the infant’s tonsil and intestinal epithelial cells (Table 1). Our findings revealed that the breast milk from most HIV-negative healthy mothers contains factors that disrupt the viral envelope and inactivate the virus. However, breast milk from nearly one-quarter of the HIV-1-negative donors exhibited no direct antiviral activity. Breast milk samples from these donors promoted HIV-1 transmission by increasing virus release from MECs and viral transcytosis via the infant’s tonsil and intestinal epithelial cells, thereby facilitating MTCT of the HIV-1 pathogen.

## 3. Materials and methods

### 3.1. Ethics statement

This study was conducted according to the principles expressed in the Declaration of Helsinki. The study was approved by the Committee on Human Research of the University of California, San Francisco (IRB approval #H8597–30664-03). All subjects provided written informed consent for the collection of samples and their subsequent analysis.

#### Viruses, viral proteins, and cells.

Laboratory-adapted dual (X4-R5)-tropic HIV-1_SF33_ and the primary isolates R5-tropic HIV-1_SF170_ and X4-tropic HIV-1_92UG029_ were grown in PBMCs activated with 2.5 μg/ml PHA (Sigma) and 1 μg/ml IL-2 (BD Biosciences) for three days. All stock viruses of HIV-1 were purified by using Amicon Ultra-15 columns as previously described (Barzegar Behrooz et al., 2019; Nazli et al., 2010). Titers of viral stocks were determined by p24 concentration based on the results of an HIV-1 p24 ELISA (PerkinElmer) performed according to the manufacturer’s instructions. The stock viruses were stored at −80 °C.

Primary tonsil epithelial keratinocytes were established from palatine tonsil tissue from HIV-negative children who were less than five years of age after routine tonsillectomy at the clinic of the Department of Otolaryngology of the University of California, San Francisco. Primary MECs were purchased from Lonza. Keratinocytes were grown in a keratinocyte growth medium (KGM gold) (Lonza) and used at early passages that were stored in liquid nitrogen.

#### Preparation of human breast milk samples and isolation of breast milk cells.

Fresh samples of human breast milk were obtained from 27 HIV-negative healthy volunteers within two to five months postpartum. The donors did not have breast inflammation or infection, and did not use drugs or alcohol. The samples were subjected to centrifugation at 600×*g* for 15 min to separate the lipid phase, milk supernatant, and cell pellets. The lipid phase was aspirated. The cell-free milk supernatant was filtered through a 0.22-μm filter, aliquoted, and stored at −80 °C. Free calcium in breast milk was measured using a colorimetric calcium assay that was performed as described in the manufacturer’s instructions (Abcam, catalog no. ab102505).

Breast milk cells (BMCs) were isolated from breast milk using a Ficoll-Paque Plus density gradient (Sigma) and frozen pending use. CD4^+^ T lymphocytes and CD14^+^ macrophages were isolated by positive selection using anti-CD4 and anti-CD14 microbeads, respectively (Miltenyi Biotec) (Tuaillon et al., 2009).

#### Establishment of polarized MECs and tonsil epithelial cells.

To establish polarized MECs, cells were grown on the lower surfaces of 3-μm Transwell^®^ two-chamber filter inserts (12-well), with the apical cell surfaces facing the lower chamber and the basal cell surfaces attached to the lower faces of the filter inserts as we described previously (Tugizov et al., 2003, 2013b). To achieve this, filter inserts were placed upside down in Petri dishes, and cells in 50 μl of medium were added to the filter surface. Inserts with cells were incubated for two to 3 h at 37 °C in 5 % CO_2_ to facilitate attachment of cells to the filters. Filter inserts were then placed in 12-well plates in the correct orientation. Attached keratinocytes prepared in this fashion formed polarized monolayers with apical membranes facing the lower chamber and the basal membrane attached to the filter insert at its lower surface. The addition of virus to the upper chambers of these inserts permitted us to evaluate virion binding to the basal surface of polarized cells.

To establish polarized primary tonsil epithelial cells, we propagated primary keratinocytes from tonsil tissue samples as previously described (Tugizov et al., 2013b; Xiao et al., 2009). Polarized tonsil epithelial cells were then established in the upper chambers of filter inserts, also as previously described (Tugizov et al., 2003, 2011, 2013b; Yasen et al., 2017, 2018). Polarized tonsil epithelial cells prepared in this fashion will have apical membranes accessible to the virus from the upper chamber, and basal membranes attached to the filter insert facing the lower chamber.

The polarity of MECs and tonsil epithelial cells was confirmed by immunodetection of the tight junction protein, occludin, and measurements of TER (Tugizov et al., 2013b) using a Millicell ERS epithelial volt-ohm meter (Millipore Corp., Billerica, MA).

#### HIV attachment, entry, transcytosis, and exocytosis assays.

To evaluate HIV attachment, HIV-1 virions at 20 ng/insert of HIV-1 p24 per ~3 ×10^5^ cells were added to the apical (tonsil and gut epithelial cells) or basolateral (MECs) surfaces (Tugizov et al., 2013b; Yasen et al., 2018). Cells were incubated at 4 °C for 2 h and then washed three times with cold phosphate-buffered saline (PBS) (pH 7.2) and lysed with 1.0 % Triton X-100 buffer (150 mM NaCl, 10 mM Tris/HCl, pH 8.0, and a cocktail of protease inhibitors [Roche]). Surface-bound HIV-1 was measured by p24 ELISA.

To assess HIV-1 internalization, polarized tonsil cells and MECs were incubated with 20 ng/insert of HIV-1 p24 on the apical and basolateral surfaces, respectively, for 2 h at 37 °C in 5 % CO_2_ (Tugizov et al., 2013b; Yasen et al., 2018). Cells were washed with PBS, pH 7.2, and treated with 0.25 % trypsin to remove extracellular virions remaining on the surface (Herrera et al., 2016). Cells were lysed with 1.0 % Triton X-100, and intracellular virus was evaluated quantitatively by HIV-1 p24 ELISA. The cutoff value for the p24 ELISA was 3.5 pg/mL, as indicated by the manufacturer (PerkinElmer).

HIV-1 transcytosis assays were performed as previously described (Tugizov et al., 2003, 2013b; Yasen et al., 2018). We confirmed cell polarization before each experiment and again after the transcytosis assay by measuring TER. For the transcytosis assay, 20 ng/insert of HIV-1 p24 from cell-free virions was added to the apical surfaces of the polarized tonsil cells or the basolateral surfaces of MECs. Cells were incubated at 37 °C in 5 % CO_2_ for 4 h. Culture media were collected from the lower chambers to evaluate HIV-1 p24 by ELISA.

For the HIV exocytosis assay, HIV-1 was added to the apical (polarized tonsil epithelial cells) or basolateral (polarized MECs) surface of the cells (Tugizov et al., 2013b; Yasen et al., 2018). After a 4-h incubation, breast milk was added to apical (MECs) or basolateral (tonsil) surfaces, followed by incubation for another 2 h. Released virions were collected (culture media/breast milk) and evaluated quantitatively with the p24 ELISA assay.

#### Measurement of SYT7 docking toward plasma membranes.

Polarized tonsil epithelial cells were incubated with breast milk or culture media containing 3 mM calcium for 30 min; untreated cells were served as a control. Cells were then fixed and immunostained for SYT7 (green) and occludin (red). Merged RGB images were used to determine the areas exhibiting simultaneous expression of both SYT7/occludin; the threshold was determined, and yellow color was selected to indicate co-localization. To determine the percentage of all cells within the colocalized area, the threshold was adjusted to select all cells within the merged RGB image; the selected area was then evaluated using the ImageJ Analyze Menu. The degree of SYT7 docking to the plasma membrane was presented as the percentage of the total area representing co-localization.

#### Transfection of cells with siRNAs.

The siRNAs for SYT7 were purchased from Santa Cruz Biotechnology. Unrelated (scrambled) siRNAs were used as controls. Polarized tonsil cells were transfected with siRNAs using lipid-based transfection reagents as previously described (Kamioka et al., 2010; Tugizov et al., 2011), and siRNA-mediated silencing of genes of interest was evaluated at 72 h after transfection on a Western blot assay probed with anti-SYT7.

#### Assessing HIV-1 sequestration in polarized epithelial cells.

Polarized epithelial cells were incubated with 20 ng/insert of HIV-1 p24 on their basolateral surface of MECs at 37 °C in 5 % CO_2_ for 4 h. After 4 h, virions remaining on the cell surface were removed with mild 0.05 % trypsin for four to 5 min at room temperature (Tugizov et al., 2012). The integrity of cell polarity after trypsin treatment was confirmed by TER measurements that were performed throughout the experiment. Cells were cultured for 1, 2, 3, and 4 days. For examination of intracellular virions, cells were treated with 0.25 % trypsin. The concentration of intracellular virus was then determined by ELISA p24.

#### Establishment of polarized-oriented tonsil and intestinal tissue explants.

Tonsil tissues without visible inflammation were collected from HIV-negative children under five years of age who were undergoing procedures to correct obstructive sleep apnea. Approximately 50–70 % of these patients exhibit no tonsil inflammation (Chan and Cistulli, 2009; Ng et al., 2005; Ngiam and Cistulli, 2015; Sutherland et al., 2012).

Small intestinal (jejunal) tissues were obtained from fetuses of HIV-uninfected women with normal pregnancies before elective termination for nonmedical reasons. Approval for the collection of infant and fetal biopsy tissues was obtained from the Institutional Review Board at the University of California, San Francisco. The tissues were placed in a tube with 2 ml of RPMI medium containing 10 % heat-inactivated fetal bovine serum, 20 mM HEPES, 100 mM glutamine, 20 μg/ml gentamicin, 200 U/ml penicillin, and 200 μg/ml streptomycin.

Explants of ~5 × 5 mm dissected from tonsil and gut epithelium that contained lamina propria were placed with the mucosal side facing up in the upper chambers of Millicell filter inserts (12-mm diameter and 3-μm pore size) (Millipore). The lateral edges of the explants were sealed with 3 % agarose, as previously described in our work (Herrera et al., 2021; Tugizov et al., 2012, 2013a). HIV-1 virions at 100 ng/insert of HIV-1 p24 cells were added to the mucosal (apical) surface of tonsil and gut epithelial tissues. After 2 h, cells and tissues were washed three times and maintained in culture medium. After each experiment, tonsil and gut tissue explants were examined by immunostaining for occludin to confirm the integrity of tight junctions. Tissues with disrupted junctions were excluded from further study.

#### Cell viability assays.

Breast milk cells or tonsil epithelial cells were seeded in wells of clear flat-bottom 96-well plates (Corning, catalog: 3595) at a density of 3 × 10^5^ or 2 × 10^5^ cells per well, respectively. CellTiter-Glo^®^ 2.0 assays were performed following the manufacturer’s instructions (Promega). Control wells containing medium alone without cells were used to determine background luminescence levels. After equilibrating the plate and its contents to room temperature for approximately 30 min, 100 μL of CellTiter-Glo^®^ 2.0 Reagent was added to 100 μL of medium with or without cells. The plate was placed on an orbital shaker for 2 min to induce cell lysis and then at room temperature for an additional 10 min to stabilize the luminescence signal. Triplicate 50 μL samples were then transferred to white flat-bottom 96-well plates (Costar, catalog no. 3917). Luminescence was measured using a Promega Glomax^®^ machine.

#### Treatment of cells and tissues with activators and inhibitors of PKC.

To activate PKC, cells were treated for 1 h with 10 μM PMA or ionomycin (Sigma-Aldrich). To inhibit PKC phosphorylation and activation, cells were treated for 2 h with 10 nM of the pan-PKC inhibitor, Go 6983 (Sigma-Aldrich).

#### Immunofluorescence assays.

Polarized epithelial cells were fixed with 4 % paraformaldehyde in PBS for 15 min, permeabilized with 0.01 % Triton X-100 in 4 % paraformaldehyde for 5 min, and infiltrated with 2 % sucrose. Tonsil explants were fixed in 3 % paraformaldehyde, infiltrated with 5–15 % sucrose, embedded in optimal cutting temperature compound, and frozen in liquid nitrogen, as previously described (Tugizov et al., 2012). The following antibodies were used for detection of HIV-1 and cellular proteins: mouse anti-HIV-1 p24 (5 μg/ml) (NIH AIDS Reagent Program); mouse anti-CD4, anti-CD68(1 μg/ml of each) (all from BD Biosciences); mouse anti-occludin (1 μg/ml) (Invitrogen); rabbit pan-anti-PKC (Invitrogen); and rabbit anti SYT7 (Abcam). Secondary antibodies labeled with DyLight 488, DyLight 594, and Alexa Fluor were purchased from Jackson ImmunoResearch. Cell nuclei were counterstained with TO-PRO-3 iodide or 4′,6-diamidino-2-phenylindole (Molecular Probes). The specificity of each antibody was confirmed by negative staining with the corresponding primary isotype control antibody. Cells were analyzed by using a Leica SP5 confocal laser microscope (Leica Microsystems) or a Nikon Eclipse E400 fluorescence microscope (Nikon).

For quantitative evaluation of HIV p24-expressing immune cells, sections were co-immunostained for viral proteins and immune cell markers (CD45 for white blood cells, CD68 for macrophages, CD4 for lymphocytes). HIV-positive immune cells were counted in the epithelium and lamina propria in at least 10 randomly selected microscopic fields (x200) per section in at least three sections for each explant. Results are presented as the average number of positive cells per mm^2^.

#### Western blot assay.

Proteins were extracted from cells with 1 % Triton X-100 buffer (150 mM NaCl, 10 mM Tris/HCl, pH 8.0) containing a cocktail of protease inhibitors (Roche) and were separated on a sodium dodecyl sulfate-polyacrylamide gel prepared with a 4–20 % gradient. Mouse monoclonal antibodies ID6 and #24–2 (from the NIH AIDS Research and Reference Reagent Program) were used to detect HIV gp120 and p24, respectively. Rabbit anti-SYT7 (Abcam) was used to detect synaptotagmin 7. PKC activation was measured by the detection of phosphorylated forms using a pool of rabbit pan anti-phospho-PKC antibodies (Thr497, Invitrogen; Ser660, Cell Signaling). Rabbit pan anti-PKC (Invitrogen) was used to detect total PKC. Equivalent protein loading was confirmed on blots probed with anti- β-actin mouse monoclonal antibody (mAb) (Ambion).

#### Statistical analysis.

Statistical comparisons were performed using two-tailed Student’s t-tests and the non-parametric Wilcoxon rank-sum test with p-values <0.05 considered significant. Results are presented as mean ± SD.

## 4. Results

### The impact of breast milk on HIV-1 infectivity in breast milk cells (BMCs) and PBMCs.

**Fig. 1.**
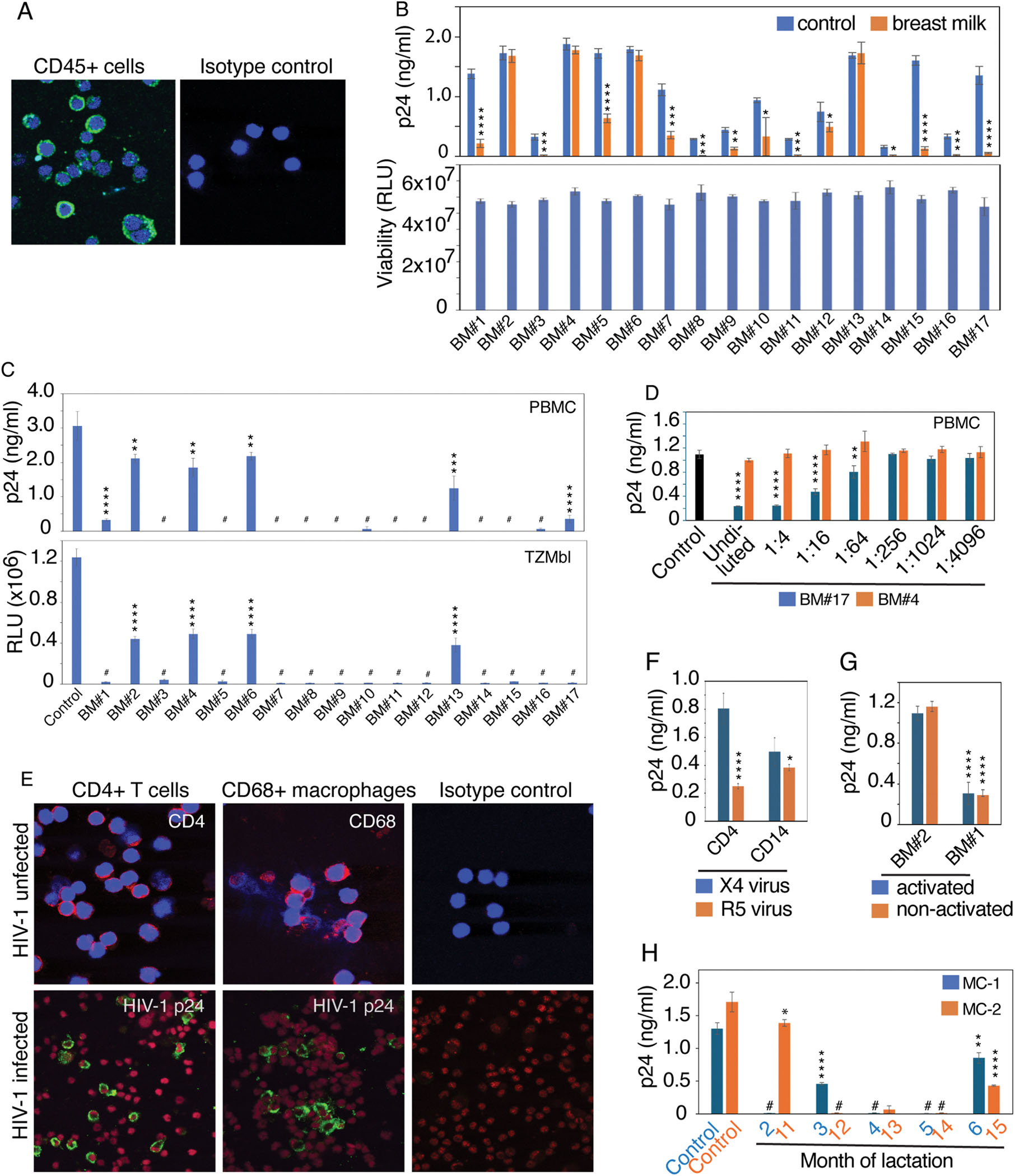
Breast milk cells are susceptible to HIV-1_SF33_ infection. (A) Breast milk cells (BMC) from donor BM#2 were immunostained with anti-CD45 (left panel) or isotype control antibodies (right panel). Nuclei were stained in blue with TO-PRO-3. Cells were analyzed by confocal microscopy; magnification × 600. (B) HIV-1_SF33_ was incubated in the undiluted clear phase of breast milk from 17 donors for 30 min at 37 °C. Virus/breast milk samples were then diluted 1:20 in culture media and used to infect matching BMCs (2 ng HIV-1_SF33_ p24 for 10^5^ cells). One set of cells was infected with an identical concentration of virus that had been incubated in culture medium (control). One set of breast milk-treated BMCs was examined for viability using an MTT assay (lower panel). Cells were washed after 2 h of incubation and resuspended in fresh media. Cells were maintained for 10 days, and HIV-1 p24 was detected in culture media using a p24 ELISA assay (upper panel). (C) PBMCs and CD4^+^-TZM-bl cells were incubated with virus/breast milk and virus/culture media (control) as described in (B). Two and ten ng HIV p24 were used to infect 10^6^ PBMCs and 10^5^ TZM-bl cells, respectively. Cells were washed, and virus infectivity was examined at 10 and 2 days postinfection by p24 ELISA (upper panel) and luciferase assay (lower panel), respectively. (D) HIV-1_SF33_ was added into diluted and undiluted breast milk samples from donors BM#4 and BM#17 and was incubated for 30 min at 37 °C. PBMCs were then infected with virus/breast milk dilutions, and cells were examined for infection by p24 ELISA after 10 days. (E and F) CD4^+^ T lymphocytes and macrophages were isolated from BM#6 and seeded into eight-chamber slides at 2 × 10^5^ cells per well (E, upper panel). After 24 h, one set of CD4^+^ T cells and one set of CD14^+^ macrophages were immunostained with anti-CD4 and anti-CD68 antibodies, respectively, and analyzed by confocal microscopy (E, upper panels; magnification × 600). The next set of CD4^+^ T cells and CD14^+^ macrophages were infected with HIV-1_SF170_ and HIV-1_UGANDA_ (2 ng p24 per 10^5^ cells). After five days, cells were fixed and immunostained with anti-HIV-1 p24 (E, lower panels; magnification × 400). One set of virus-infected cells was maintained for 10 days and then examined by p24 ELISA assay (Fig. 1F). (G) One set of BMCs was activated with PHA and IL2 for three days, while one set of cells remained untreated. These cells were then infected with HIV-1_SF33,_ and viral infection was examined by p24 ELISA 10 days later. (H) Breast milk samples were collected from donors MC#1 and MC#2 each month from two to six and 11–15 months of lactation, respectively. Anti-HIV activity was examined in BMCs isolated from breast milk from donor BM#13. HIV-1_SF33_ was incubated in breast milk for 30 min. Then, BMCs were infected with virus/breast milk from donor MC#1 or from donor MC#2. Twelve days later, the cells were examined by p24 ELISA assay. Data in panels B, C, D, F, G, and H are shown as mean ± standard deviation (SD) (n = 3) and represent findings from two independent experiments; *P < 0.05, **P < 0.01, ***P < 0.001, and ****P < 0.0001, in comparison with the control groups. #, not detected.

To examine the contributions of breast milk to the infectivity of cell-free HIV-1, samples were collected from 17 clinically healthy HIV-negative women. All samples in this first set were mature (i.e., they did not contain colostrum) and were collected during the first two to five months of lactation. From these breast milk samples, BMCs were isolated. Immunostaining of BMCs with anti-CD45 revealed that nearly 100 % of the isolated cells were WBCs (Fig. 1A). Dual tropic HIV-1_SF33_ virions were mixed with the undiluted clear phase of breast milk, incubated at 37 °C for 30 min, and then used to infect the matching BMCs (i.e., BMCs isolated from the same breast milk sample) (Fig. 1B), PBMCs isolated from the blood of HIV-negative individuals, and cells of the CD4^+^ TZM-bl reporter line. After ten days in culture, HIV-1_SF33_ infection of BMCs and PBMCs was examined by HIV-1 p24 ELISA. HIV-1_SF33_ infection of the TZM-bl cells was examined after 48 h using a luciferase expression assay.

Our analysis revealed that 13 of 17 breast milk samples (76 %) completely or partially inhibited HIV-1 infection in matching BMCs. By contrast, breast milk samples from four donors (BM#2, BM#4, BM#6, and BM#13) did not prevent HIV-1 infection of matching BMCs. Incubation of HIV-1_SF33_ virions with BM#2, BM#4, BM#6, and BM#13 for various time points, including 15, 30, 45, and 60 min, did not change the viral infectivity of PBMCs (data not shown). The results of a cell viability assay confirmed that more than 90 % of the BMCs from all donors were alive after completion of the experiment (Fig. 1B, lower panel).

In a parallel experiment, HIV-1 infection of PBMCs revealed that breast milk either completely inhibited (11 donors) or substantially reduced (2 donors) the extent of HIV-1 infection (Fig. 1C, upper panel). The four samples identified as inactive in the matched BMC assay (Fig. 1B) elicited partial reductions in HIV-1 infection in these experiments. Further analysis revealed that HIV-1 virions incubated with breast milk from the same 13 donors elicited no luciferase activity in TZM-bl cells (Fig. 1C, lower panel). The remaining four breast milk samples partially inhibited luciferase activity (approximately 50–60 %) in this assay.

To confirm and further examine their potential anti-HIV activity (or lack thereof), breast milk samples from donors BM#4 and BM#17 were evaluated in dilution assays with PBMCs (Fig. 1D). Results showed that the antiviral effect of the BM#17 sample was gradually reduced upon dilution. By contrast, the BM#4 sample exhibited no antiviral effects and no inhibition at any dilution.

To examine infection of X4-and R5-tropic HIV-1 infection of BMCs, breast CD4^+^ T lymphocytes and macrophages were isolated by positive selection using anti-CD4 and anti-CD14 microbeads, respectively. Subsets of the CD4^+^ T cells and macrophages were immunostained with anti-CD4 and anti-CD68, respectively. Our results revealed that more than 95 % of T cells and macrophages were CD4^−^ and CD68-positive, respectively, thereby confirming their phenotypes (Fig. 1E, upper panels). Additional subsets of CD4^+^ T cells and macrophages were infected with dual-tropic HIV-1_SF33_. Five days later, the cells were immunostained with anti-HIV-1 p24. Confocal microscopy revealed that ~30 % of the CD4^+^ T cells and ~15 % of the macrophages were p24-positive (Fig. 1E, lower panels). Another set of cells was maintained for 10 days, and viral infection was examined by p24 ELISA. Our findings revealed that both CD4^+^ T cells and CD14^+^ macrophages were infected by both X4 and R5 tropic viruses, although p24 levels were higher in cells infected with the former strain (Fig. 1F). Breast milk macrophages and CD4 T lymphocytes have been shown to express CD4, CCR5, and CXCR4 (Kourtis et al., 2007; Lee et al., 2025; Satomi et al., 2005), which may facilitate infection of both HIV-1 R5 and X4 strains.

BMCs are frequently described as activated cells (Field, 2005; Hsu and Nanan, 2018; Ichikawa et al., 2003; Lewis et al., 2017; Tuaillon et al., 2009). To explore the impact of exogenous activation, BMCs isolated from breast milk samples from donors BM#1 and BM#2 were treated with 2.5 μg/mL phytohemagglutinin (PHA) and 1 μg/mL IL-2 for three days, while additional sets of cells from these same donors were left untreated. PHA/IL-2 and untreated cells were then infected with HIV-1_SF33_ virions that had been preincubated with matching BM#1 (antiviral) and BM#2 (non-antiviral) breast milk samples. Our results revealed similar infections in both PHA/IL-2-treated and control cells (Fig. 1G). Thus, our findings confirm the activation status of BMCs and reveal that the impact of breast milk on HIV-1 infection does not rely on additional activation.

Finally, we examined the antiviral activity of breast milk collected at multiple times from two donors with more than several months of experience with lactation. The samples were collected from donor MC#1 from the second to the sixth month (earlier lactation), and from donor MC#2 from the 11th through the 15th month of lactation (later lactation). The antiviral effects of each breast milk sample were examined using BMCs from donor BM#13. The data revealed that breast milk from donor MC#1 collected at three and six months of lactation reduced HIV-1 infection by approximately 65 % and 30 %, respectively (Fig. 1H). Similarly, samples from donor MC#2 collected at 11 and 15 months of lactation reduced HIV-1 infection by approximately 25 % and 70 %, respectively. Breast milk samples collected from both donors at all other timepoints yielded complete virus inactivation. We concluded that the antiviral activity of breast milk was not stable during the entire lactation period. During extended periods of lactation, breast milk from a single donor may at times completely and at times partially inactivate the virus, thereby opening a window permitting infection of HIV-susceptible cells.

### Breast milk disrupts HIV-1 envelope gp120 and inhibits viral attachment to CD4^+^ T cells.

**Fig. 2.**
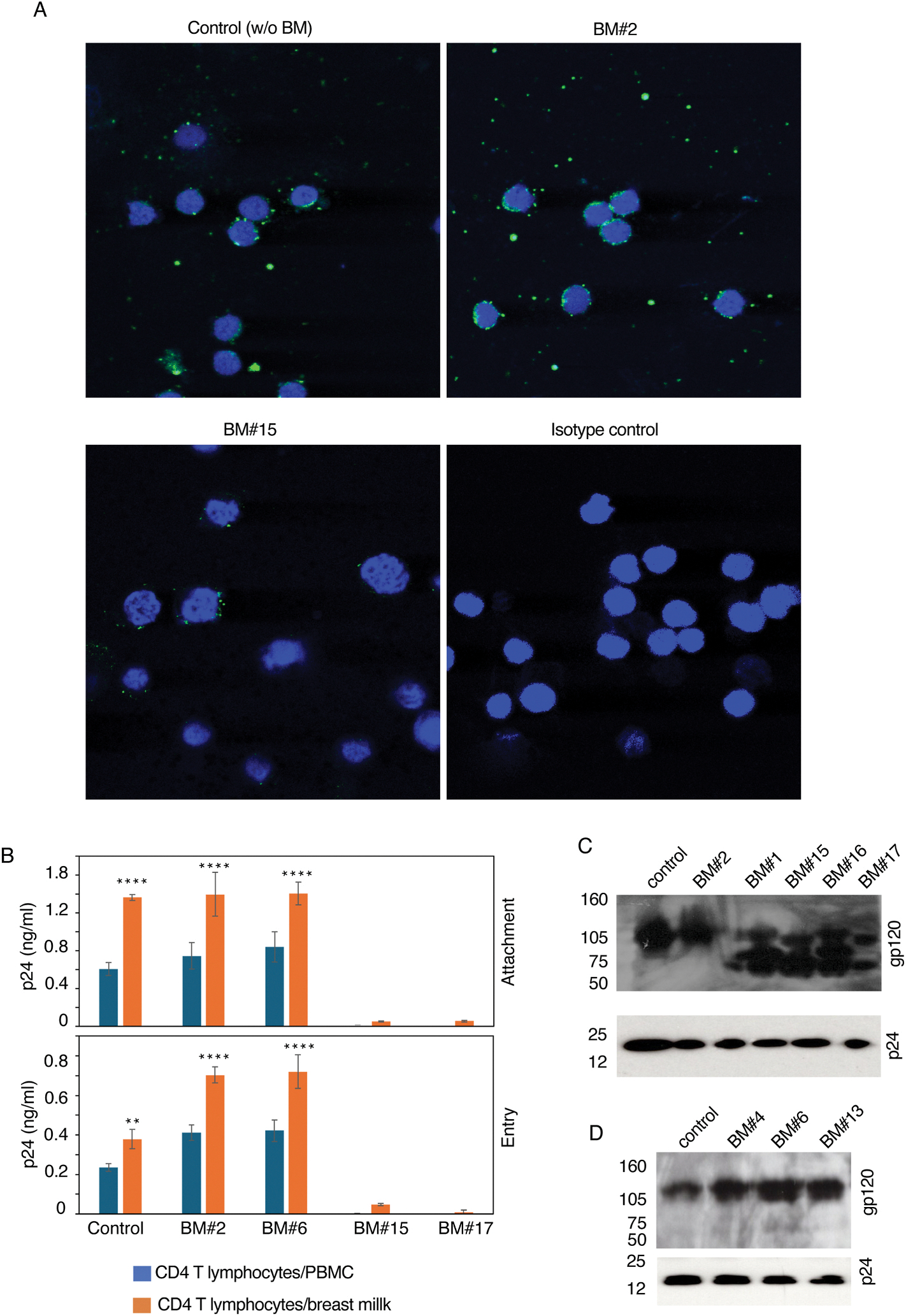
Pre-incubation with breast milk prevents HIV-1 binding to the surface of CD4^+^ T lymphocytes. (A) CD4^+^ T cells were isolated from breast milk samples from donor BM#2. HIV-1_SF33_ was preincubated with BM#2 and BM#15 for 30 min at 37 °C. Virus/breast milk samples were then cooled on ice for 10 min and added to CD4^+^ T cells at 10 ng per 10^6^ cells, followed by incubation for 2 h at 4 °C (on ice). Cells were then washed, fixed, and immunostained with anti-p24 antibody. Nuclei were stained blue with TO-PRO-3, and cells were analyzed by confocal microscopy (magnification × 800). (B) HIV-1_SF33_ was left untreated or incubated in breast milk samples from donors BM#2, BM#6, BM#15, and BM#17 for 30 min at 37 °C and then used in quantitative attachment and entry assays. CD4^+^ T cells isolated from BMCs from donor BM#2 and PBMCs were left untreated or infected with HIV-1_SF33_ pre-incubated with breast milk samples at 10 ng per 10^6^ cells. To examine virus attachment, cells were incubated with HIV-1_SF33_ at 4 °C for 2 h, then washed and lysed. Cell surface-bound virions were detected by p24 ELISA. To examine virus entry, the HIV-1_SF33_ was added to the cells and incubated for 2 h at 37 °C. After incubation, the cells were treated with 0.25 % trypsin to remove the extracellular virus. Intracellular virus was detected with p24 ELISA. Data are shown as mean ± SD (n = 3) and represent findings from three independent experiments; **P < 0.01 and ****P < 0.0001 compared to controls. (C and D) HIV-1_SF33_ was left untreated or incubated with breast milk samples from donors BM#2, BM#1, BM#15, BM#16, and BM#17 (C), and BM#4, BM#6, and BM#13 (D) for 30 min at 37 °C. Samples were evaluated on Western blots probed with anti-HIV-1 gp120 and anti-p24. The data shown represent findings from two independent experiments.

To understand the mechanisms underlying its antiviral effects, we examined the binding of HIV-1_SF33_ to CD4^+^ T lymphocytes isolated from breast milk BM#2. The virus was preincubated in breast milk samples from donors BM#2 (no antiviral activity) and BM#15 (with antiviral activity; see Fig. 1) for 30 min and then added to cells on ice to examine viral attachment or at 37 °C to facilitate entry. Analysis of virus attachment by confocal microscopy revealed virion binding to cell surfaces of both controls (no preincubation with breast milk) and samples that were pretreated with breast milk from donor BM#2, (Fig. 2A). By contrast, only a few virions were detected on the surfaces of cells incubated with virions pretreated with breast milk from donor BM#15.

To evaluate virus binding and entry quantitatively, cells were washed, and both cell surface-bound and internalized virions were examined by p24 ELISA. In this experiment, both CD4^+^ T cells isolated from breast milk and from PBMCs were used for attachment and entry experiments. Our results revealed that virus attachment was twofold higher in CD4^+^ T cells isolated from breast milk compared to those isolated from PBMCs (Fig. 2B). Pre-incubation of virus with breast milk from donors BM#2 or BM#6 had no discernible impact on viral attachment and led to a significant increase in virus entry into CD4^+^ T cells from both sources (Fig. 2B).

As shown, breast milk samples from donors BM#15 and BM#17 completely inhibit virus binding and entry in CD4^+^ T lymphocytes isolated from breast milk and PBMCs (Fig. 2B). These findings suggest that breast milk may contain factors that promote disruption of the viral envelope. To test this possibility, purified HIV-1_SF33_ virions were incubated in samples of undiluted breast milk that did (donors BM#1, BM#7, BM#15, and BM#17) or did not (BM#2) exhibit antiviral effects. After 30 min, viral proteins gp120 and p24 were examined by Western blot assay. Two to three immunoreactive fragments were detected with anti-gp120 antibody in samples containing virus pretreated with breast milk from donors BM#1, BM#7, BM#15, or BM#17 (Fig. 2C). In contrast, only a single, higher molecular weight band was detected in the sample treated with breast milk from donor BM#2 and in the untreated control. Breast milk samples BM#4, BM#6, and BM#13, which demonstrated no anti-viral effects, also showed a single band of gp120 (Fig. 2D).

Collectively, these data indicated that breast milk samples with antiviral activity might potentially damage the viral envelope glycoprotein gp120, thereby preventing viral attachment and penetration into CD4^+^ T lymphocytes. In contrast, breast milk samples with no apparent antiviral activity did not change or damage the viral envelope, or affect virion binding and entry. Of note, pretreatment of the virus with breast milk did not decrease the size of HIV-1 p24, thus suggesting that the capsid protein remained unchanged.

### Basolateral to apical transport of HIV-1 through polarized MECs.

**Fig. 3.**
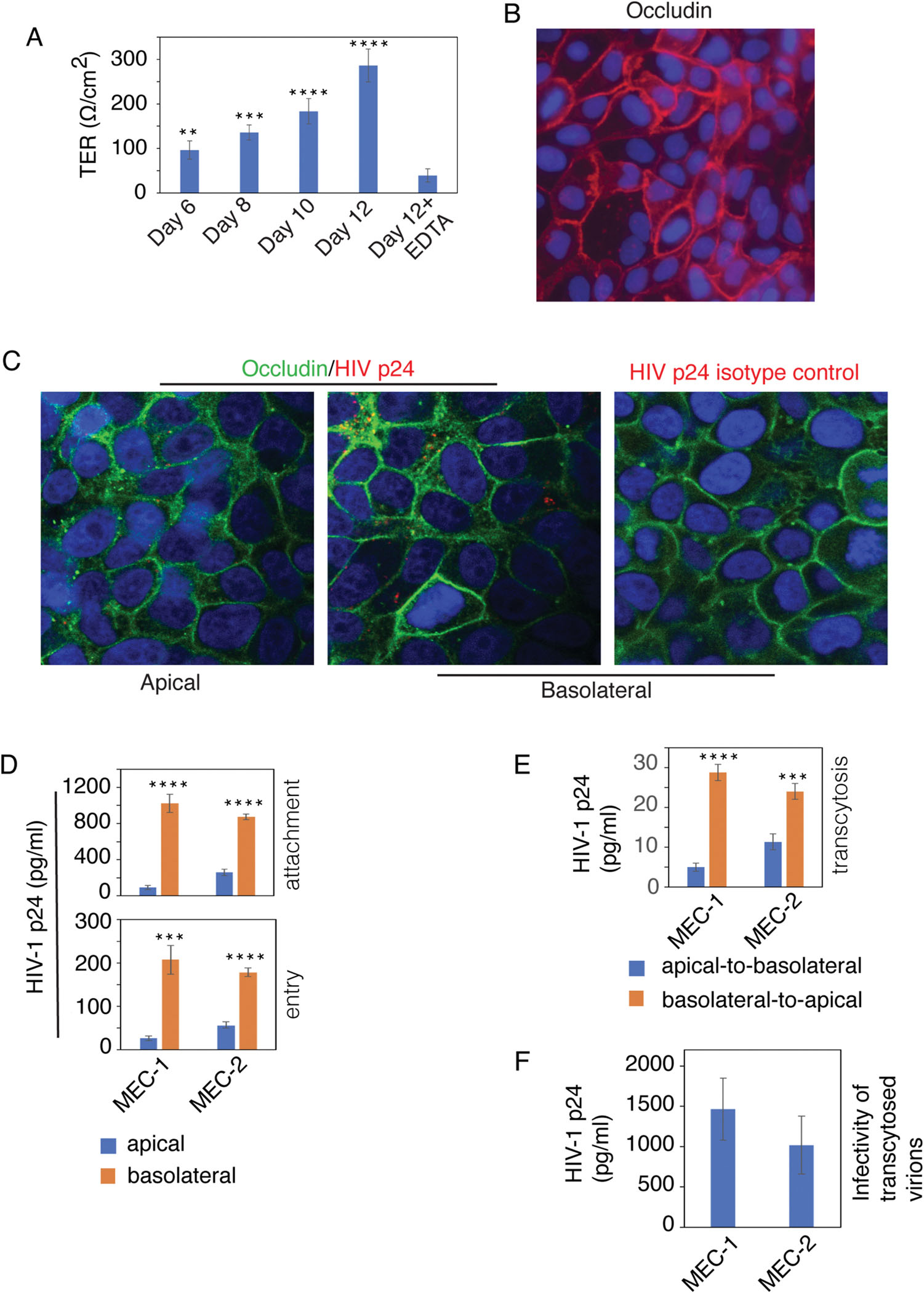
Basolateral-to-apical transcytosis of HIV through polarized MECs. (A) Polarized MECs were established using two-chamber Transwell^®^ inserts. TER of polarized cells was measured from day 6 through day 12. At day 12, one set of cells was incubated with 10 mM EDTA for 30 min, and the TER was measured. Data are shown as mean ± SD (n = 3). The results are representative of two independent experiments; **P < 0.01, ***P < 0.001, and ****P < 0.0001 compared to the EDTA-treated depolarized cells. (B) Cells at day 12 were immunostained for tight junction protein, occludin, and analyzed by confocal microscopy. Cell nuclei were stained blue with TO-PRO-3; magnification × 600. (C) HIV-1_SF33_ was added to the apical or basolateral surfaces of MECs from two different donors (MEC-1 and MEC-2). After 30 min, intracellular virus was detected by immunostaining with anti-HIV p24 (red) and anti-occludin (green) by confocal microscopy. Cell nuclei were stained blue with TO-PRO-3; magnification × 600. (D and E) HIV-1_SF33_ attachment and entry (D) and transcytosis from apical and basolateral membranes (E) of polarized MECs from two independent donors evaluated by p24 ELISA. (F) MECs from two independent donors were used to evaluate basolateral-to-apical transcytosis of HIV-1_SF33_. Transcytosed virus was collected and used to infect BMCs isolated from BM#13. After 12 days, viral infectivity was evaluated with p24 ELISA. (D, E, and F) *P < 0.05, ***P < 0.001, and ****P < 0.0001 compared with apical attachment, entry, and transcytosis. These data are representative of three independent experiments using MECs from two different donors.

Polarized MECs were established using primary mammary keratinocytes from two independent donors. The transepithelial resistance (TER) of polarized cells in culture gradually increases over time. On day 12, the TER of MEC cultures reached ~300 Ω/cm^2^ (Fig. 3A). At this time point, one set of polarized cells was incubated with 10 mM ethylenediamine tetraacetic acid (EDTA) for 30 min. EDTA treatment disrupted tight junctions and resulted in a drastic reduction of TER (Fig. 3A). Immunostaining of the tight junction protein occludin revealed its localization in the lateral membranes in a ring pattern (Fig. 3B). Collectively, these features confirm MEC polarization *in vitro*.

We examined the entry of dual-tropic HIV-1_SF33_ into the apical or basolateral membranes of polarized MECs. While HIV-1_SF33_ virions were detected in dot-like vesicular patterns on both membranes by confocal microscopy, intracellular virus was detected more readily in MECs infected via their basolateral membranes (Fig. 3C). Quantitative analysis of HIV-1_SF33_ attachment and entry into MECs with p24 ELISA confirmed that these events occurred predominantly at the basolateral membranes (Fig. 3D). Viral transcytosis also proceeds predominantly in a basolateral-to-apical direction (Fig. 3E). Virions that underwent transcytosis in MECs were capable of infecting mononuclear cells incubated with breast milk samples from donor BM#13 (Fig. 3F).

### Breast milk induces the release of sequestered virions from polarized MECs.

**Fig. 4.**
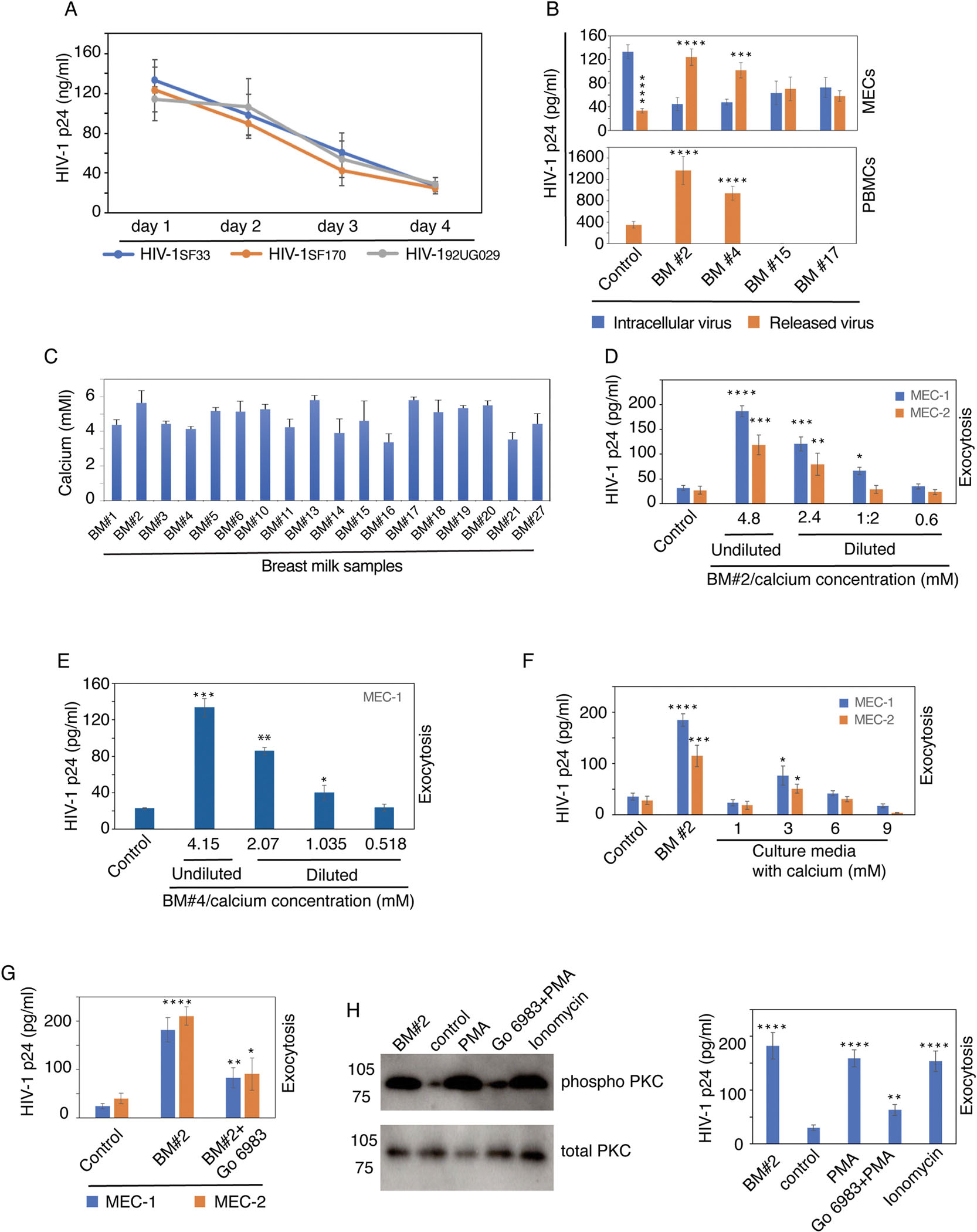
Breast milk induces HIV-1 exocytosis from polarized MECs. (A) HIV-1_SF33_, HIV-1_SF170,_ or HIV-1_92UG029_ (20 ng/ml p24) were added to the upper chamber of Transwell^®^ inserts (i.e., the basolateral surface of polarized MECs) and the cells were incubated for 4 h. Virions remaining on the surface of the basolateral membranes were then removed with trypsin, and the cells were maintained in culture for 1, 2, 3, and 4 days. At each time point, intracellular virions were detected using a p24 ELISA. (B) HIV-1_SF33_ was added to the basolateral surface of polarized MECs, and cells were incubated for 4 h. Virions remaining on the membrane surface were removed with trypsin, and cells were cultured for an additional 24 h. Culture media from the lower/apical chamber was removed, and undiluted breast milk samples from donors BM#2, BM#4, BM#15, and BM#17 were added to this chamber. Control groups were left untreated. After 2 h, culture media from the lower chambers were collected and assayed for released virus using a p24 ELISA (upper panel). Intracellular virus was detected by p24 ELISA (upper panel). PBMCs were infected with released virus, and after ten days HIV-1 infection was examined using p24 ELISA assay (lower panel). (C) Free calcium ions were measured in the clear phase of breast milk samples from 18 donors using a colorimetric calcium assay kit. (D, E, and F) HIV-1_SF33_ virions were added to the basolateral surface of polarized MEC-1 and MEC-2 cells. After 24 h of incubation, culture media from the lower/apical chambers were then replaced with undiluted or diluted (1:2, 1:4, and 1:8) breast milk from donor BM#2 (D) or BM#4 (E) and incubated for an additional 2 h. The concentration of calcium in undiluted and diluted breast milk samples is shown. In parallel experiments, media from the lower/apical chambers were replaced with fresh media containing 1, 3, 6, or 8 mM calcium (F). Untreated cells served as the control. After 2 h, media from the lower/apical chambers were collected and evaluated quantitatively by p24 ELISA. (G) Experiments with MEC-1 and MEC-2 were performed as described in (D). One set of cells was incubated with Go 6983 for 2 h. Media from the lower/apical chambers were replaced with breast milk from donor BM#2; the cells pretreated with Go 6983 were treated again with this inhibitor. After 2 h, media were collected and assayed for released virus using a p24 ELISA. (H) Polarized MEC-1 cells were exposed to HIV-1_SF33_ from the basolateral surface for 4 h, and media from the lower/apical chambers were replaced with breast milk from donor BM#2 or media containing PMA, Go 6983, or ionomycin. After 2 h, culture media were collected from the lower/apical chambers and examined for released virus by p24 ELISA (right panel). Cells were then lysed and evaluated by Western blot to detect both total and phosphorylated PKC (left panel). (A–G) Results are shown as means ± SD (n = 3); *P < 0.05, **P < 0.01, ***P < 0.001, and ****P < 0.0001 compared with appropriate control cultures. (E) Results are shown as means ± SD (n = 3); *P < 0.05, **P < 0.01, and ***P < 0.001, compared with control cells. (A–H) Results were reproduced in two independent experiments.

We showed previously that most of the HIV-1 virions internalized into polarized tonsil and genital epithelial cells were sequestered in endosomal compartments (Yasen et al., 2017, 2018). To determine if this was also the case for MECs, polarized cells were exposed to dual-tropic HIV-1_SF33_, R5-tropic HIV-1_SF170_, and X4-tropic HIV-1_92UG029_ via their basolateral membranes. After 4 h, cells were washed to remove virions that remained on the surface, and cultures were maintained for 1, 2, 3, and 4 days thereafter. Each day, one set of cells was dissociated with trypsin, and intracellular virus was evaluated quantitatively by p24 ELISA. Our data revealed that all three virus strains were sequestered in the epithelial cells for up to four days (Fig. 4A). Although the concentration diminished gradually over time, intracellular virus (at approximately 40 pg/ml) remained detectable at day four post-inoculation.

*In vivo*, the apical surfaces of MECs are in contact with breast milk. Breast milk contains significant concentrations of calcium, which may play a role in inducing exocytosis of sequestered intracellular viruses. To evaluate the impact of breast milk and calcium on viral exocytosis, HIV-1_SF33_ was added to the basolateral surface of polarized MECs, and cells were maintained for 24 h. MECs were then incubated for 2 h with breast milk samples from donors BM#15 and BM#17 (with antiviral activity) and donors BM#2 and BM#4 (no antiviral activity). Culture medium with breast milk was collected from the apical compartment to detect the released virus. Cells were then dissociated with trypsin, and intracellular virus was evaluated by p24 ELISA. Our findings revealed that approximately 70 % virus was intracellular (i.e., sequestered) in control cells (Fig. 4B, upper panel) and only 30 % of the virus was released from apical membranes (i.e., had undergone transcytosis after its introduction at the basolateral membranes). By contrast, most (70 %) of the virus detected by p24 ELISA was released from apical membranes in MECs treated with breast milk from donors BM#2 and BM#4; these results suggested that breast milk might promote viral exocytosis. Samples from donors BM#15 and BM#17 induced the release of approximately 50 % of the total virus. This may be because breast milk samples with antiviral activity can inactivate virus released into the apical compartment and limit the quantity that can be recovered in this assay.

To examine the infectivity of the released virions, culture medium collected in this experiment was used to infect PBMCs; after 10 days, virus infection was examined by p24 ELISA (Fig. 4B, lower panel). Our data revealed that virions released from untreated control cells and cells treated with breast milk samples from donors BM#2 and BM#4 remained infectious (i.e., remained capable of infecting PBMCs). By contrast, virions released from MECs treated with BM#15 and BM17 were not infectious, an observation that may also be related to the direct antiviral effects observed in these breast milk samples. Collectively, these findings suggest that there are factors in breast milk that induce viral exocytosis from the apical membranes of MECs regardless of their antiviral functions.

To understand the mechanism of breast milk-induced HIV exocytosis, we examined the concentration of free calcium in 18 breast milk samples. We found that the calcium concentration varied from ~4 to 6 mM (Fig. 4C). Addition of the calcium chelator, EDTA, to the breast milk samples resulted in the rapid depolarization of MECs and thus inhibition of HIV transcytosis. Given this limitation, we instead reduced the free calcium concentration by diluting the breast milk samples (Fig. 4D and E). We found that dilution of breast milk samples BM#2 and BM#4 with calcium-free media reduced the concentration of calcium and HIV release (Fig. 4D and E), consistent with the potential role of calcium in promoting viral exocytosis. We also compared HIV exocytosis in MECs treated with breast milk from donor BM#2 and culture media containing 1, 3, 6, and 9 mM calcium. A higher level of HIV exocytosis was detected in culture medium containing 3 mM calcium (Fig. 4F), which is approximately one-half to three-quarters of the calcium concentration found in our breast milk samples. These results suggested that breast milk may contain other factors that facilitate HIV exocytosis.

Calcium-dependent activation of PKC may play a critical role in exocytosis. To evaluate this possibility, polarized MEC-1 and MEC-2 cells were pretreated with the PKC inhibitor, Go 6983, an intervention that reduced breast milk (donor BM#2)-induced HIV-1_SF33_ exocytosis by approximately 50 % (Fig. 4G). PKC activation and breast milk (donor BM#2)-induced viral exocytosis was then tested in cells treated with phorbol 12-myristate 13-acetate (PMA), a biochemical that induced both PKC phosphorylation (Amos et al., 2005) (Fig. 4H, left panel) and HIV exocytosis to an extent comparable to that observed in response to breast milk from donor BM#2 (Fig. 4H, right panel). Cells pretreated with Go 6983 blocked both PMA-induced PKC phosphorylation and HIV exocytosis. Finally, treatment of cells with ionomycin, a calcium ionophore that activates PKC by increasing intracellular calcium levels, also induced both PKC phosphorylation and HIV exocytosis. Collectively, these findings suggest that high concentrations of calcium in breast milk can induce HIV exocytosis from MECs.

### Breast milk increases transcytosis of HIV-1 through polarized infant tonsil epithelial cells by activating calcium-dependent PKC.

**Fig. 5.**
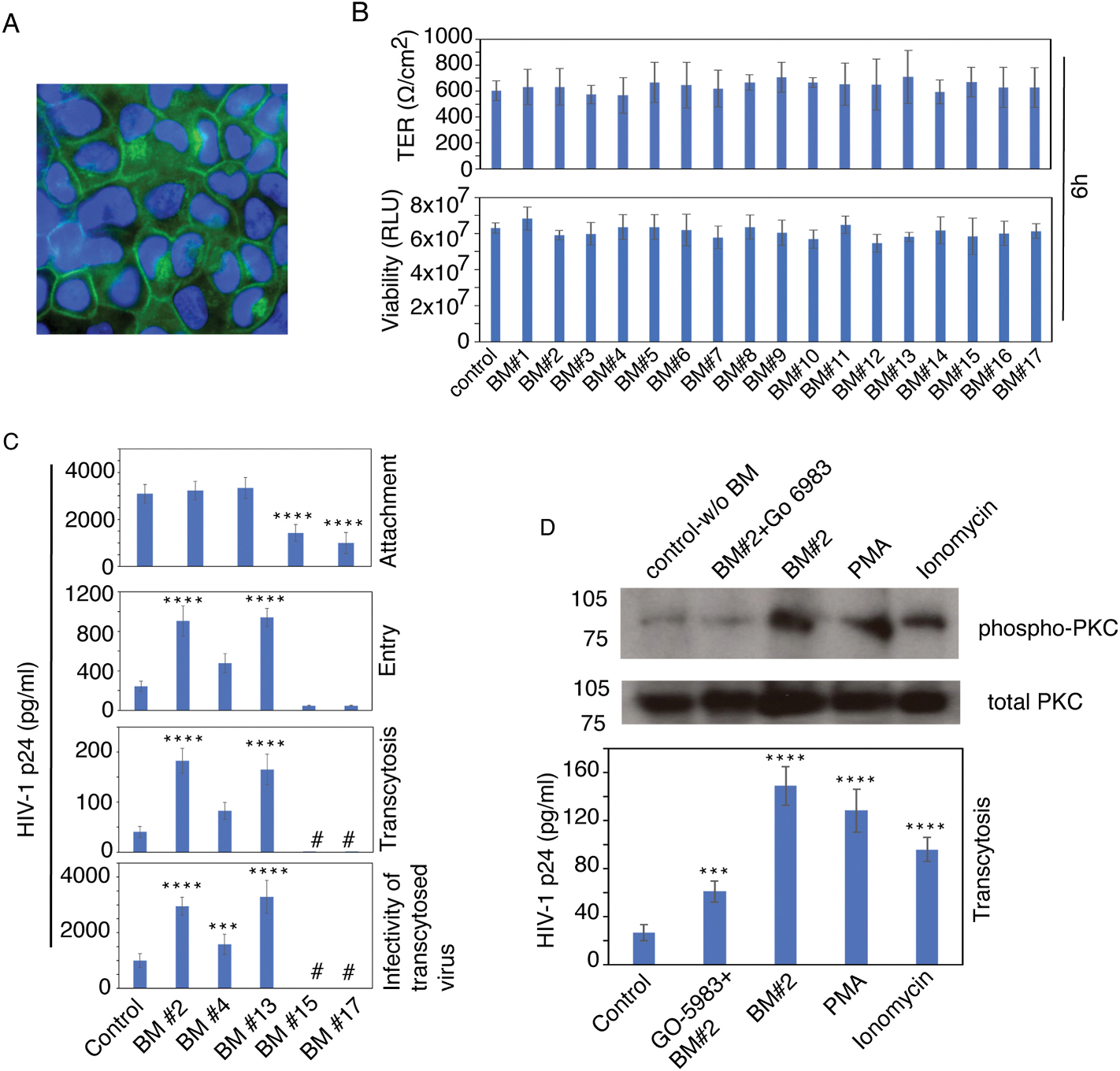
Breast milk promotes HIV-1 transcytosis through polarized infant tonsil epithelial cells. (A) Polarized tonsil epithelial cells were established on Transwell^®^ inserts. On day 14, cells were immunostained with antibodies that recognize the tight junction protein, occludin and analyzed by confocal microscopy. Cell nuclei were stained blue with TO-PRO-3; magnification × 600. (B) Polarized tonsil epithelial cells were incubated with undiluted breast milk from donors BM#1 – BM#17 for 6 h at 37 °C. TER of the polarized cells was then evaluated using a Millicell-ERS volt-ohm meter (upper panel). Next set of cells were examined for cell viability using CellTiter-Glo^®^ 2.0 assay (lower panel). (C) HIV-1_SF33_ attachment, entry, and transcytosis were examined in polarized tonsil epithelial cells in the presence of breast milk from donors BM#2, BM#4, BM#13, BM#15, and BM#17. Cells without breast milk served as controls. To examine HIV-1_SF33_ attachment, cells were incubated with virus (20 ng/insert of p24) for 2 h at 4 °C. Cells were then washed, detached, and lysed, and virus was evaluated quantitatively by p24 ELISA. To evaluate virus internalization, virions were added to cells and incubated for 2 h at 37 °C. Extracellular virus was removed with trypsin, and then intracellular virus was evaluated quantitatively by p24 ELISA. Viral transcytosis was measured in culture media from the lower chambers of the Transwell^®^ inserts after 4 by ELISA after 4 h of incubation. In parallel experiments, the infectivity of transcytosed virions collected from the lower chamber was examined by targeting PBMCs. (D) To examine the role of breast milk in promoting PKC activation and HIV-1 transcytosis, cells were left untreated or treated with BM#2, BM#2 with Go 5983, PMA, or ionomycin for 1 h. One set of cells was lysed; total and phosphorylated PKC were evaluated by Western blot (upper panel). HIV-1_SF33_ transcytosis was examined in cells incubated with BM#2, BM#2 with Go 5983, PMA, or ionomycin. After 4 h, transcytosed virions were detected in the lower chamber by p24 ELISA (lower panel). (B, C, and D) Data are shown as mean ± SD (n = 3); **P < 0.01, ***P < 0.001, and ****P < 0.0001 compared with appropriate control conditions. Results were reproduced in two independent experiments.

Incubation of polarized tonsil epithelial cells (Fig. 5A) for 6 h with breast milk samples from 17 donors resulted in no change in TER or cell viability (Fig. 5B). HIV-1_SF33_ pre-incubated in breast milk samples from donors BM#15 and BM#17 (with antiviral activity) or donors BM#2, BM#4, and BM#13 (no antiviral activity) were introduced at the apical cell surface, and virus attachment, entry, and transcytosis were examined (Fig. 5C). Our results revealed that breast milk samples without anti-HIV activity (BM#2, BM#4, and BM#13) maintained equivalent HIV-1_SF33_ attachment to the apical surface of tonsil epithelial cells (Fig. 5C, upper panel). However, other breast milk samples incubated with virions with antiviral activity (BM#15 and BM#17) exhibited substantially reduced virus binding to the cell surface. Virus entry and transcytosis were also increased by two-to fourfold in cells treated with samples from donors BM#2, BM#4, and BM#13 compared to control (Fig. 5C, second and third panels from top). In contrast, no virus was identified in entry and transcytosis experiments involving breast milk samples from donors BM#15 and BM#17. The infectivity of transcytosed virus was examined in PBMC infection experiments, which showed that virus from cells treated with BM#2, BM#4, and BM#13 were infectious at rates two-to fourfold higher than in control cells. No infectious virus was detected from cell cultures treated with BM#15 and BM#17 (Fig. 5C, lowermost panel). Taken together, these findings indicate that breast milk samples with antiviral function can completely inhibit HIV-1 transcytosis via polarized tonsil epithelial cells. In contrast, breast milk samples lacking antiviral function promote viral transcytosis through tonsil epithelial cells.

To examine the functional consequences of calcium-dependent PKC activation and their impact on HIV-1 transcytosis, polarized tonsil epithelial cells were treated with breast milk from donor BM#2 (both with and without Go 6983), PMA, and ionomycin. One set of cells served as untreated controls. After 30 min, one set of cells was collected and used to detect total and phosphorylated PKC by Western blotting. A second set of cells was used to evaluate HIV-1_SF33_ transcytosis. Findings from Western blotting revealed that breast milk from donor BM#2, together with Go 6983, inhibited PKC phosphorylation by approximately 60 % compared with cells treated with breast milk from donor BM#2 alone (Fig. 5D, upper panel). This result suggests that calcium in the breast milk sample promoted PKC activation in tonsil epithelial cells. PMA and ionomycin also promote PKC phosphorylation under these conditions, confirming the role of calcium in PKC activation. Analysis of transcytosis revealed that breast milk from donor BM#2 increased HIV-1_SF33_ transcytosis six-fold compared to control cells (Fig. 5D, lower panel). Breast milk from donor BM#2 combined with Go 6983 reduced breast milk-induced viral transcytosis by approximately threefold compared to cells exposed to breast milk from donor BM#2 alone, also revealing a functional role for breast milk calcium in promoting virus transcytosis. PMA and ionomycin also increased viral transcytosis by approximately three to fourfold compared to control, suggesting that calcium-mediated activation is a critical feature of viral transcytosis.

### The calcium sensor, SYT7, promotes HIV-1 exocytosis from polarized tonsil epithelial cells.

**Fig. 6.**
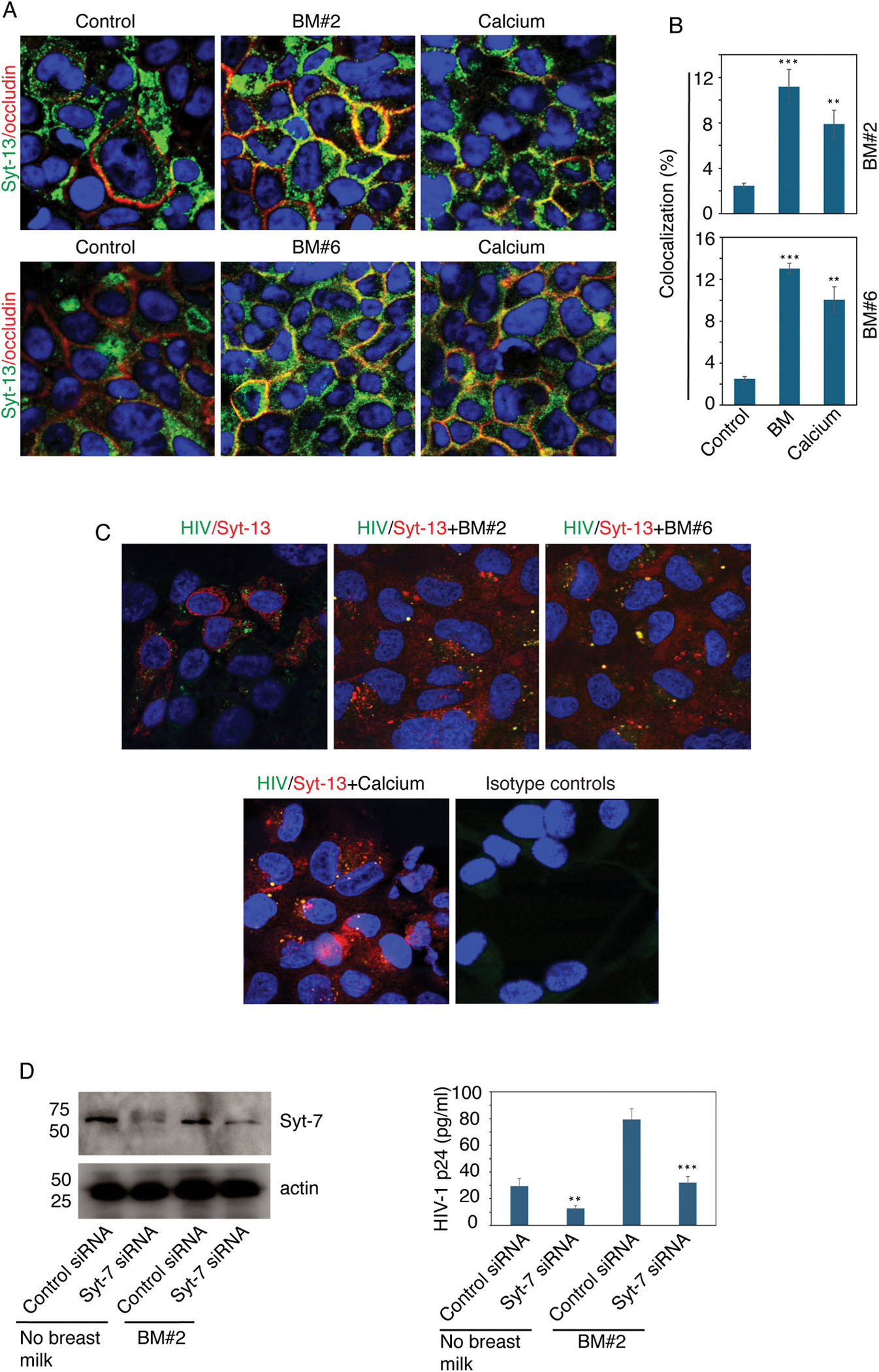
SYT7 mediates breast milk-induced HIV exocytosis from tonsil epithelial cells. (A and B) Polarized tonsil epithelial cells were incubated with breast milk from donor BM#2, BM#6, or culture media containing 3 μM calcium for 30 min; untreated cells served as controls. Cells were immunostained with anti-SYT7 (green) and anti-occludin (red). Merged images are presented, with yellow color indicating colocalization of SYT7 and occludin on the lateral membranes of polarized cells (A). Areas exhibiting SYT7 and occludin colocalization were evaluated quantitatively using Image J software and presented as % colocalization (B). (C) HIV-1_SF33_ was added to the apical surface of polarized tonsil epithelial cells. After 4 h, virions remaining on the apical surface were removed with trypsin. Cells were then incubated with breast milk from donor BM#2, BM#6, or culture media containing 3 μM calcium for an additional 30 min. Cells were then fixed and immunostained with anti-HIV-1 p24 (green) and anti-SYT7 (red). Merged images are presented, with the yellow color indicating the colocalization of the virus and SYT7. (A and C) Cell nuclei were stained blue with TO-PRO-3; magnification × 600. (D) Left panel: Polarized tonsil epithelial cells were transfected with SYT7 and control (scrambled) siRNA. SYT7 expression was examined three days later by Western blot (upper panel) with beta-actin serving as the loading control (lower panel). One set of cells was used to examine HIV-1_SF33_ transcytosis. HIV-1 was added to the apical surfaces of SYT7-silenced and control cells. After 4 h, medium in the basolateral compartment was replaced with breast milk from BM#2 or culture media and incubated for 30 min. Exocytosed virus was detected in culture medium from the lower chambers by p24 ELISA. (B and D) Data are shown as mean ± SD (n = 3); **P < 0.05, and ***P < 0.001, compared with relevant control conditions. The results are representative of two independent experiments.

The data presented in the previous sections (Figs. 4 and 5) suggested that calcium found in breast milk may play a critical role in promoting HIV exocytosis from MECs and tonsil epithelial cells, thereby reducing viral sequestration in the cells. To confirm this hypothesis, we examined the role of calcium sensor SYT7 in HIV-1 exocytosis in tonsil epithelial cells. Immunofluorescence and Western blot assays targeting SYT7 in MECs revealed no detectable expression (data not shown). However, when the same assays were used to examine tonsil epithelial cells, we found that they expressed SYT7 in both a cytoplasmic and a vesicular pattern (Fig. 6A and C). When compared to untreated cells, polarized tonsil cells incubated with breast milk BM#2 and BM#6 for 30 min experienced a shift in the cytoplasmic distribution of SYT7 from submembrane areas to the membrane, where it colocalized with occludin (Fig. 6A, middle panels). Incubation with 3 μM calcium also resulted in increased colocalization of SYT7 with occludin (Fig. 6A, right panels). By contrast, cells that were not exposed to breast milk showed no substantial colocalization of these proteins (Fig. 6A, left panels). Quantitative analysis revealed a fourfold increase in SYT7 and occludin co-localization observed in cells treated with breast milk from donor BM#2 and BM#6 compared to untreated control cells (Fig. 6B). Calcium-treated cells also showed threefold higher SYT7 and occludin co-localization than in control cells. Collectively, these data suggest that calcium at levels found in breast milk may induce docking of SYT7-containing vesicles at the plasma membrane.

Next, tonsil cells exposed to HIV-1_SF33_ were incubated for 30 min with breast milk from donor BM#2 or BM#6 or left untreated. Immunostaining of these cells with anti-HIV-1 p24 and anti-SYT7 antibodies revealed colocalization of HIV-1 and SYT7 in cells incubated with breast milk (Fig. 6C). Colocalization was not detected in control cells, which were not exposed to breast milk. These results suggest that calcium/SYT7 interactions may facilitate the fusion of HIV- and SYT7-containing vesicles, thereby initiating exocytosis via fusion with the plasma membranes. To evaluate its potential direct role in promoting HIV-1 exocytosis, SYT7 expression was silenced using an siRNA approach (Fig. 6D, left panel). HIV-1_SF33_ was added to these cells. After 4 h, one set of cells was incubated with breast milk from donor BM#2 for 30 min, while another set of cells was left untreated. Analysis of HIV release by p24 ELISA revealed that cells with siRNA-mediated suppression in SYT7 expression exhibited approximately 60 % reduction of breast milk-induced viral exocytosis (Fig. 6D, right panel), indicating the critical role of SYT7 interaction with breast milk calcium in viral exocytosis.

### Breast milk substantially increased HIV transmission through infant tonsils and intestinal epithelial cells.

In an earlier experiment, we showed that breast milk promotes HIV-1 transcytosis and exocytosis from polarized tonsil epithelial cells that had been established *in vitro*. To examine this phenomenon *ex vivo*, we established polarized-oriented tonsil and gut epithelial tissue explants from tissues of infant and fetal origin, respectively, as we described in our previous work (Tugizov et al., 2012). HIV-1_SF33_ was added to the mucosal (apical) surfaces of tissue explants together with breast milk from donors BM#6 or BM#15, and viral penetration into the epithelium and infection of target cells were examined at days 1 and 5 postinfection, respectively.

**Fig. 7.**
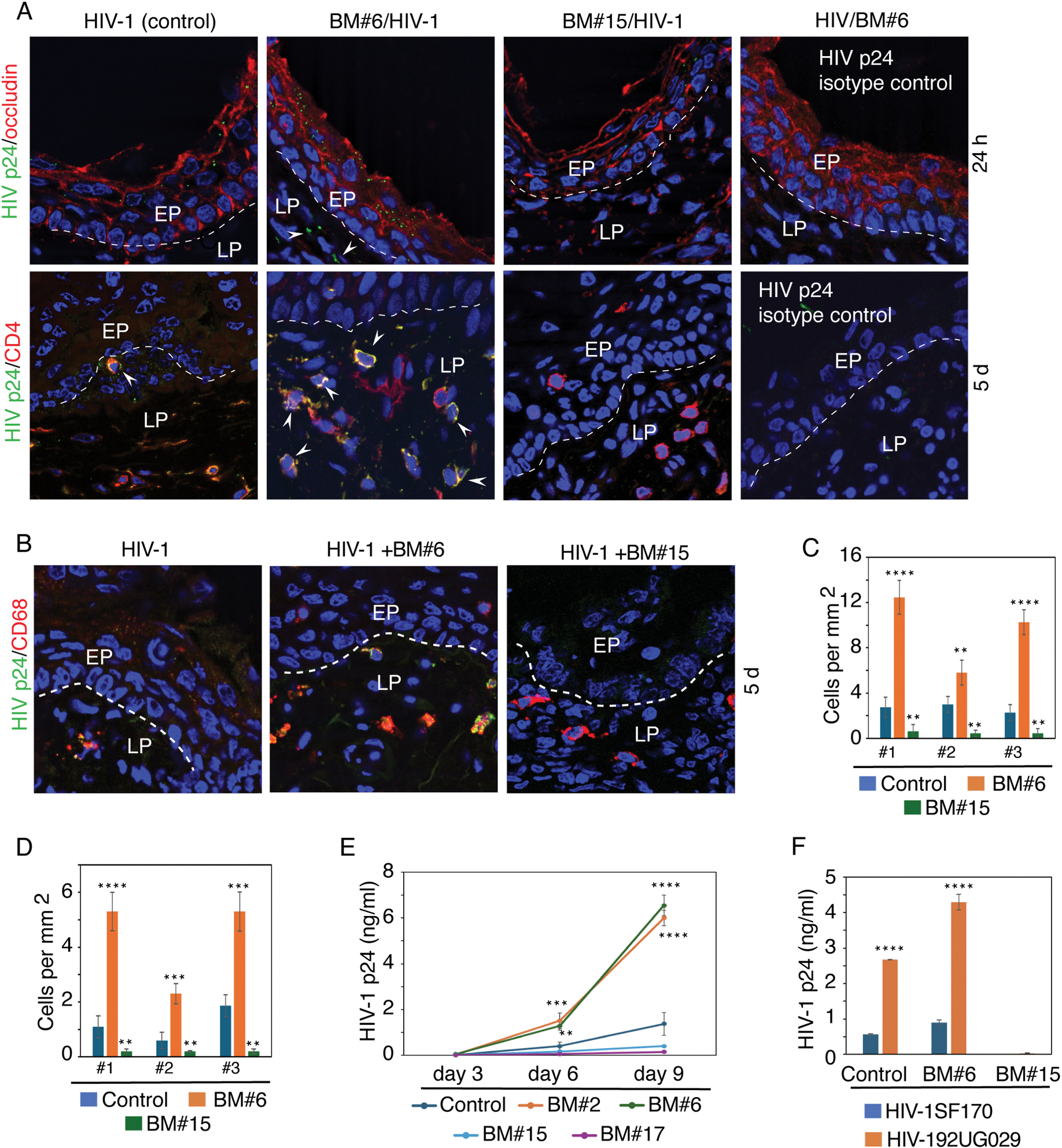
Breast milk facilitates HIV-1 transmission through the tonsil epithelium. (A) HIV-1_SF33_ was incubated with BM#6 and BM#15 for 30 min, then added to the mucosal surfaces of polarized, oriented tonsil tissue explants (in triplicate, two sets). Tissues infected with the virus alone, without breast milk served as a control. After 24 h, one set of explants was fixed and immunostained with anti-HIV-1 p24 and anti-occludin (A, upper panel). The next set of triplicated explants was maintained for an additional 5 days and coimmunostained with anti-HIV-1 p24 and anti-CD4 (A, lower panel). (B) Tissue sections from 5-day-old infections were coimmunostained with anti-HIV-1 p24 and anti-CD68. (C and D) HIV-1 p24^+^/CD4^+^ (C) and p24^+^/68^+^ (D) cells were counted (at least ten fields) from three tissue explants and are presented in cells per mm^2^. (E) HIV-1_SF33_ preincubated with breast milk BM#2, BM#6, BM#15, or BM#17 were used to infect tonsil explants in triplicate. Tissues infected with HIV-1_SF33_ without breast milk served as controls. Culture medium from the lower chambers was collected on days 3, 6, and 9 postinfection and evaluated with p24 ELISA. (F) Tonsil tissue explants were infected with X4-tropic HIV-1_92OG029_ and R5-tropic HIV-1_SF170_ with breast milk BM#6 or BM#15. Explants infected with HIV-1 in the absence of breast milk served as controls. On day 9 postinfection, the released virus was evaluated in culture medium from the lower chambers of triplicate explants with p24 ELISA. (A and B) Cell nuclei were stained blue with TO-PRO-3, and tissues were evaluated with confocal microscopy at × 400 magnification. (C, D, E, and F) Data are shown as mean ± SD (n = 3); **P < 0.01, ***P < 0.001, and ****P < 0.0001 compared with controls infected without breast milk. These data are representative of two independent experiments using tonsil tissues from two different donors.

**Fig. 8.**
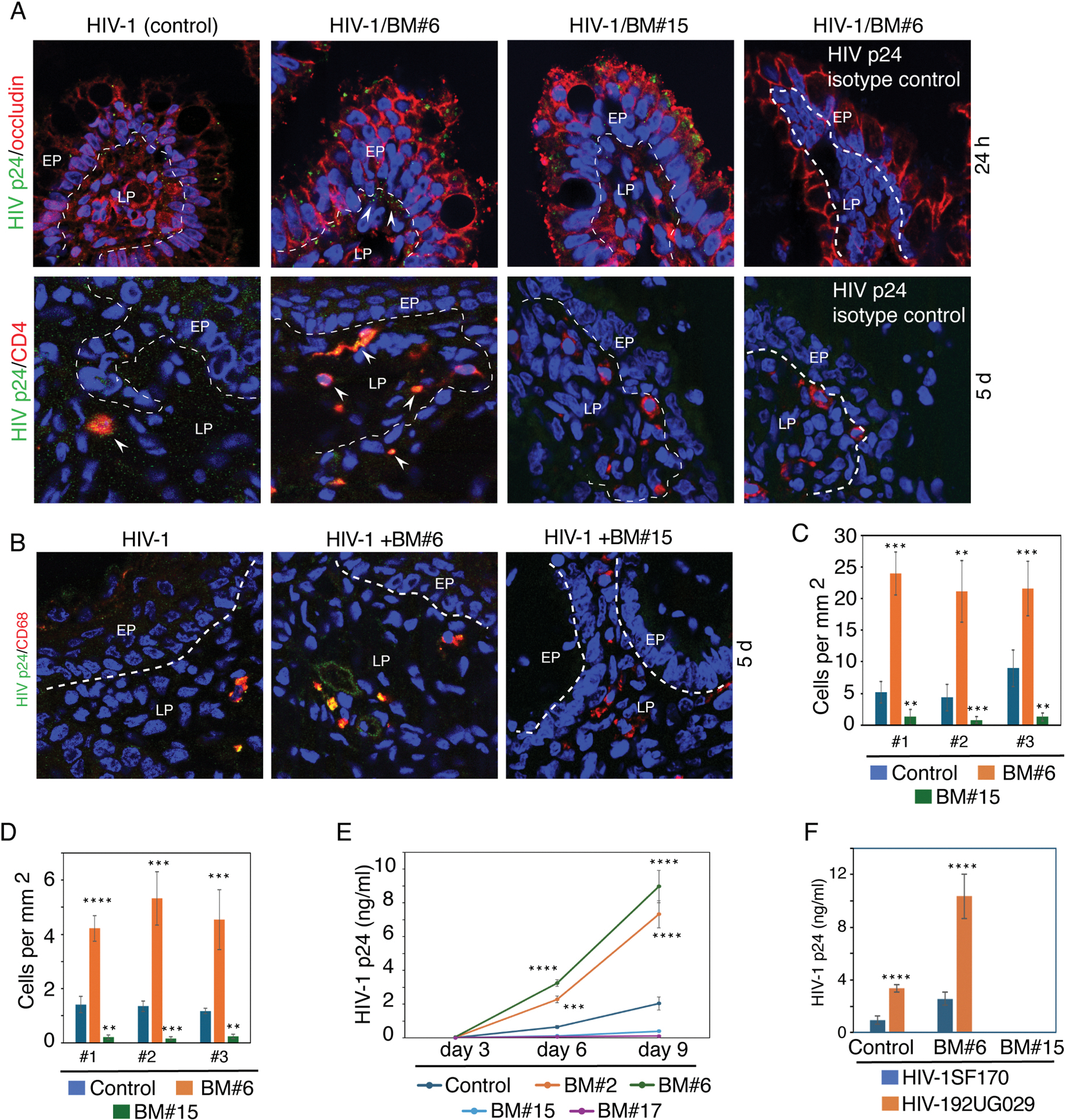
Breast milk facilitates HIV-1 transmission through the intestinal epithelium. (A and B) HIV-1_SF33_ pretreated with breast milk samples from donors BM#6 and BM#15 was added to the mucosal surfaces of polarized, oriented triplicate intestinal tissue explants. After 24 h, one set of tissues was fixed and immunostained for HIV-1 p24 and occludin (A, upper panel). Another set of tissues was cultured for an additional 5 days and coimmunostained for HIV-1 p24 and CD4 (A, lower panel) or p24 and CD68 (B). Cell nuclei were stained blue with TO-PRO-3, and the tissues were analyzed with confocal microscopy at × 400 magnification. (C and D) HIV-1 p24^+^/CD4^+^ and HIV-1 p24^+^/CD68^+^ cells were counted in at least ten fields. Average counts are presented from three tissue explants. (E) Intestinal tissue explants were infected with HIV-1_SF33_ with BM#2, BM#6, BM#15, or BM#17. Tissues exposed to HIV alone without breast milk served as the control. Virus was detected in culture medium from the lower chambers on days 3, 6, and 9 postinfection by p24 ELISA. (F). Intestinal tissue explants were infected with X4-tropic and R5-tropic HIV-1 with breast milk BM#6 or BM#15. Tissues infected without breast milk served as a control. On day 9 postinfection, culture medium from the lower chambers was tested for released virus by p24 ELISA. (B, D, E, and F) Data are shown as mean ± SD (n = 3). **P < 0.01, ***P < 0.001, and ****P < 0.0001 compared with control cells without breast milk. Results were reproduced in two experiments using intestinal tissues from independent donors.

The results of these experiments revealed that the virus penetrated the stratified tonsil epithelium, and that some of the virions reached the lamina propria during 24-h incubation period (Fig. 7A, upper panels). Virus was also detected in the epithelial cells and lamina propria of the intestinal epithelium (Fig. 8A, upper panels). More virus was detected in tissues treated with breast milk from donor BM#6, a sample with no direct antiviral activity. By contrast, little to no virus was detected in epithelial cells in tissues treated with antiviral breast milk from donor BM#15.

Confocal microscopy analysis of HIV infection of target cells at day 5 postinfection indicated that BM#6 facilitated HIV-1 infection of subepithelial CD4^+^ T cells and CD68^+^ macrophages in both tonsil and gut epithelial tissues (Fig. 7A, lower panel; Fig. 7B; Fig. 8A, lower panel; and Fig. 8B), in contrast to control tissues. Quantitative evaluation of HIV-1_SF33_-infected CD4^+^ and CD68^+^ cells in tonsil and intestinal epithelia revealed five-to sixfold more infected cells in tissues treated with breast milk from donor BM#6 than in control tissues not treated with breast milk (Fig. 7C and D, Fig. 8C and D). No HIV-infected cells were detected in tissues treated with breast milk from donor BM#15, consistent with complete inhibition of virus transmission.

To examine viral release, we added HIV-1_SF33_ to the apical surfaces of tissue explants together with breast milk from donors BM#2, BM#6, BM#15, or BM#17. Samples were tested for virus on days 3, 6, and 9 postinfection from the subepithelial compartment. Results from p24 ELISA data revealed no detectable virus on day 3 (Figs. 7E and 8E); viral detection gradually increased over days 6 and 9 postinfection. Virus release on day 9 from tissues treated with BM#2 and BM#6 was approximately five-to sixfold higher than that detected in control tissues. No virus release was detected from tissues treated with breast milk from donors BM#15 or BM#17, indicating that breast milk samples with antiviral activity completely inhibit HIV transmission via tonsil and intestinal epithelia.

Release of X4-tropic HIV-1_92UG029_ and R5-tropic HIV-1_SF170_ viruses was then examined in tonsil and intestinal tissues pretreated with breast milk from donors BM#6 and BM#15. Cells pretreated with breast milk from donor BM#6 released substantially more virus overall, while cells pretreated with breast milk from donor BM#15 inhibited the release of both viruses (Figs. 7F and 8F). The results of these experiments confirmed that breast milk samples with and without antiviral activity can inhibit or promote viral infection, respectively.

**Fig. 9.**
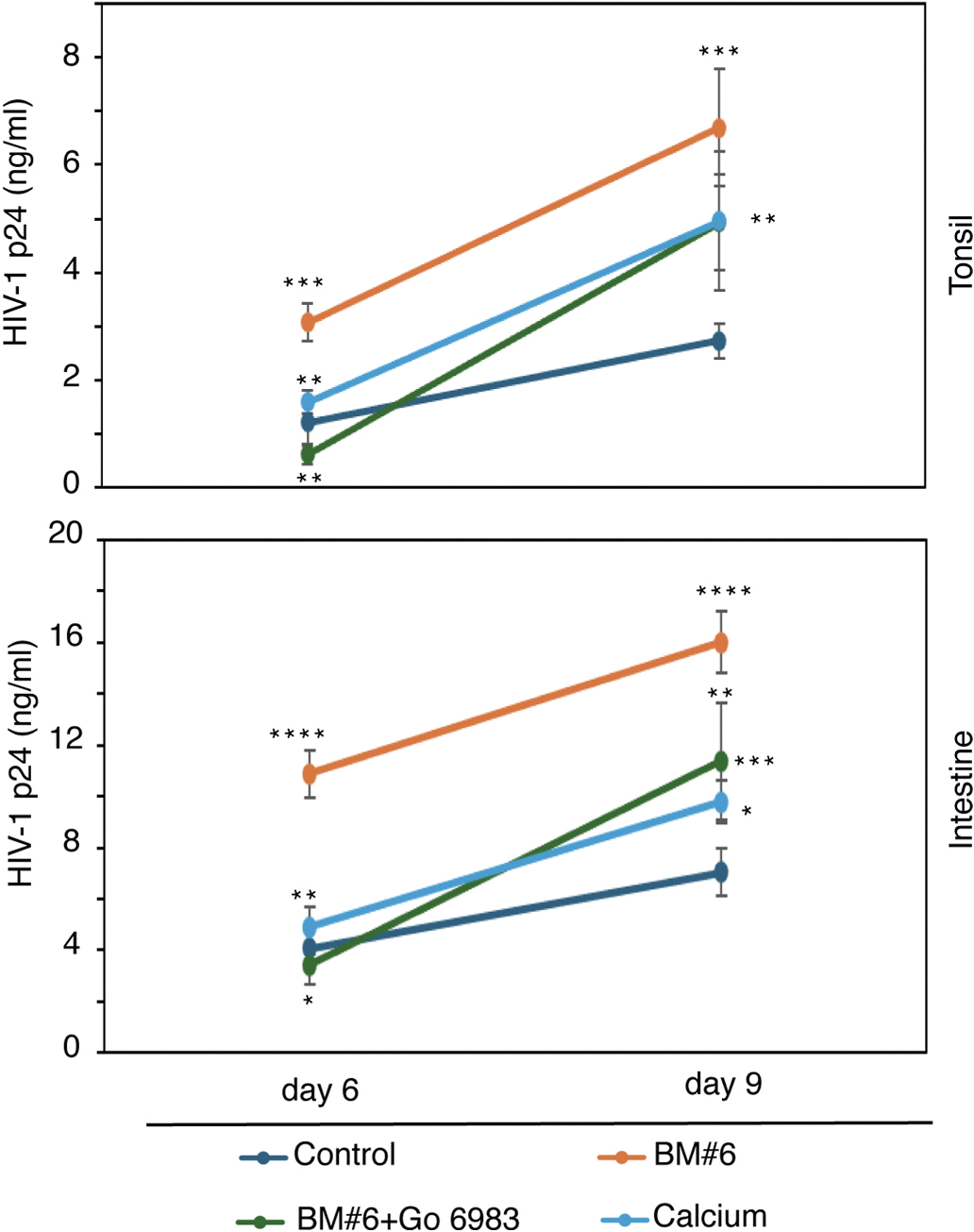
Inhibition of PKC reduces breast milk-induced HIV-1 transmission via tonsil and intestinal epithelium. Polarized, oriented tonsil and intestinal epithelial explants were treated for 2 h with the PKC inhibitor Go 5983. HIV-1_SF33_ in breast milk BM#6 or media containing 3 mM calcium was added to the mucosal surface of the polarized intestinal tissue explants, including tissues pretreated with Go 5983. After 24 h, tissues were washed and maintained for five days. Culture medium from the lower chambers was examined for HIV-1 p24 using an ELISA assay. Data are shown as mean ± SD (n = 3); *P < 0.05, **P < 0.01, ***P < 0.001, and ****P < 0.0001 compared with control cells without breast milk and without Go 5983. The results represent two experiments using independent tissue donors.

To study the contributions of breast milk-induced activation of PKC in facilitating HIV −1 transcytosis/exocytosis in tonsil and intestinal tissues, tissues were exposed to HIV-1_SF33_ in the presence of breast milk from donor BM#6 (both with and without the PKC inhibitor, Go 5983), calcium, and virus only. Detection of released virus by p24 ELISA revealed that virus infection induced by breast milk from donor BM#6 was diminished in the presence of the PKC inhibitor in both tonsil and intestinal tissues (Fig. 9). The results of this experiment suggest that activation of PKC by calcium present in breast milk may promote increased HIV-1 endocytosis, transcytosis, and exocytosis within the mucosal epithelium, and thus facilitate infection of HIV-1 target cells both within and beneath the epithelium. The increase in viral infection observed in response to calcium treatment is consistent with this interpretation.

## 5. Discussion

We have shown here that breast milk samples from 76 % of our donors can completely or partially inhibit HIV-1 infection of breast milk BMCs and prevent viral transcytosis through infant tonsil mucosal epithelium. By contrast, breast milk samples from 24 % of our donors exhibited no direct antiviral activity and also increased the rate of HIV-1 exocytosis from mammary epithelial cells and viral transcytosis through infant tonsil and gut epithelial cells, both of which play an important role in facilitating MTCT of HIV.

Productive HIV-1 infection of CD4^+^ T lymphocytes and CD68^+^ macrophages in breast milk (Field, 2005; Hsu and Nanan, 2018; Ichikawa et al., 2003; Lewis et al., 2017; Tuaillon et al., 2009) represents a significant source of both cell-free and cell-associated viruses that can initiate MTCT at this locale. Some breast milk samples contain factors that protect against HIV MTCT. In this study, breast milk samples with antiviral activity were found to contain factors causing HIV-1 gp120 dysfunction, thereby inhibiting viral attachment, entry, and transmission. Among the factors that may target gp120, breast milk contains several proteases, including furin, plasmin, carboxypeptidase B2, thrombin, aminopeptidase, kallikrein, elastase, and cathepsin D, B, H and S (Dallas et al., 2014, 2015a, 2015b; Guerrero et al., 2015; Holton et al., 2014). All or some of these enzymes may degrade viral glycoproteins, leading to conformational change and damage (Demers-Mathieu et al., 2018). HIV-1 gp120 has eight cleavage sites recognized by cathepsins L, S, and D; six of these cleavage sites are localized within receptor binding regions of gp120 (Yu et al., 2010). Protease-mediated processing and cleavage at one or more of these sites might cause structural changes and dysfunction in gp120 that prevent viral binding to its receptor (i.e., CD4) expressed on T cells.

Breast milk also contains numerous innate antiviral proteins, including lactoferrin, mucin-1, secretory leukocyte protease inhibitor, lysozyme, tenascin C, soluble toll-like receptor-2, and defensins (Baricelli et al., 2015; Jia et al., 2001; Wedekind and Shenker, 2021). These proteins may bind to HIV-1 membranes and glycoproteins, leading to structural damage and virus inactivation (Baricelli et al., 2015; Jia et al., 2001; Wedekind and Shenker, 2021). Several groups have reported that lactoferrin can bind directly to the V3 loop of HIV-1 gp120 (Farquhar et al., 2008; Habte et al., 2006, 2007, 2008a, 2008b, 2010; Harmsen et al., 1995; Henrick et al., 2012; Liu and Newburg, 2013; Newburg, 2013; Schoen et al., 1997; Swart et al., 1996, 1998). Furthermore, both lactoferrin and lysozyme inhibit HIV-1 binding to the CD4 receptor on T cells, potentially via interactions occurring at the viral envelope (Behbahani et al., 2018). Others have reported that tenascin C binds to the viral envelope and neutralizes viral infectivity (Fouda et al., 2013; Mangan et al., 2019). Similarly, human beta-defensins 2 and 3 might bind the HIV-1 envelope in breast milk and disrupt gp120, thereby leading to gp120 degradation (Herrera et al., 2021).

We also observed higher levels of HIV-1 attachment and entry in CD4^+^ T lymphocytes isolated from breast milk than in analogous cells isolated from PBMCs. These results suggest that the many cytokines and chemokines contained in breast milk, for example, IL-1β, IL-2, IL-4, IL-6, and TNF-α (Aknouch et al., 2022; Baricelli et al., 2015; Bottcher et al., 2000; Brenmoehl et al., 2018; Jia et al., 2001; Kielbasa et al., 2021; Morniroli et al., 2021; Nolan et al., 2019; Schocker and Jappe, 2022; Wedekind and Shenker, 2021), may induce target cells to express higher levels of HIV-1 receptors (Lyimo et al., 2009).

The unstable antiviral effect observed in breast milk samples collected during periods of prolonged lactation might be due to a relative increase or decrease in the concentration of proteases and other antiviral proteins. Results from several targeted studies revealed that protein, carbohydrate, and lipid concentrations in breast milk may change during lactation (Czosnykowska-Lukacka et al., 2018, 2019; Kulesza-Bronczyk et al., 2023; Lehman et al., 2019). For example, breast milk samples from women experiencing prolonged lactation (longer than 18 months) exhibit increased concentrations of lipid and protein and decreased concentrations of carbohydrates compared with samples from women who have been lactating for less than 12 months. Breast milk composition might also change significantly, reflecting the diet of the lactating mother (Bravi et al., 2016, 2021; Di Maso et al., 2021, 2022; Moro et al., 2019). Thus, the concentration of antiviral proteins in breast milk might change during lactation. Breast milk from a single donor might at one time completely inactivate HIV, and at another, exhibit little to no antiviral activity. More longitudinal studies are required to understand the role of breast milk in HIV inactivation during the prolonged lactation period.

We also found that breast milk samples with no direct antiviral activity induced a significant increase in HIV-1 entry into CD4^+^ T cells. This observation led us to consider the possibility that calcium in breast milk might induce viral endocytosis. Numerous earlier studies have documented HIV entry into T cells via endocytosis (Clotet-Codina et al., 2009; de la Vega et al., 2011; Marin et al., 2019; Miyauchi et al., 2009; Permanyer et al., 2010; Sharma et al., 2023).

Vectorial transport of HIV-1 from basolateral to apical membranes of MECs may explain the initial step of HIV MTCT. While MECs do not support productive HIV-1 infection, the results of co-cultivation experiments indicate that these cells can facilitate viral endocytosis and passage to CD4^+^ T lymphocytes (Dorosko and Connor, 2010). However, these experiments were performed by using non-polarized MECs, and thus do not provide any insight into viral transcytosis through polarized cells, a feature that is critical to our understanding of viral transmission from circulation to the breast milk of HIV-1-infected mothers. MECs express galactosylceramide (GalCer) (Dorosko and Connor, 2010), which may facilitate virus binding and endocytosis from the basolateral surface of polarized cells (Yasen et al., 2018). Transcytosis of HIV-1 in basolateral-to-apical direction via polarized MECs is the initial step in viral MTCT via breast milk.

Most virions are sequestered in the MECs, as was reported earlier in studies focused on oral and genital epithelial cells (Yasen et al., 2017, 2018). Elevated calcium concentrations in breast milk and calcium-induced PKC activation may contribute prominently to viral exocytosis, which is the final stage of viral transcytosis through MECs. However, viral exocytosis induced in response to treatment with calcium alone in culture media was much less effective than that resulting from exposure to breast milk. These results suggest that breast milk may contain additional factors, for example, calcium sensors and calcium-binding proteins that may contribute to viral exocytosis. Similarly, calcium-dependent PKC activation in breast milk may play a key role in promoting HIV exocytosis from MECs. PKC promotes and coordinates multiple events, including the disruption of cortical actin-cytoskeleton and the docking and fusion of vesicles at the plasma membranes, all actions that ultimately lead to exocytosis (D’Amico et al., 2021; Moussa et al., 2025; Yang et al., 2013). In vivo, breast milk remains in close contact with the apical surface of MECs; thus, HIV-1 sequestration in MECs should be much less significant than in the oral and genital epithelium (Yasen et al., 2017, 2018).

Despite the comparatively high concentrations of calcium in all breast milk samples evaluated, HIV-1 exocytosis induced by those with antiviral activity was substantially lower than exocytosis induced by samples with no antiviral effects. This suggests that calcium is unlikely to be the only factor inducing viral exocytosis. One possibility is that breast milk samples with significant antiviral effects may be missing one or more additional molecules that are critical for promoting viral exocytosis. Furthermore, virions released in response to breast milk with anti-HIV effects were not infectious in PBMCs. This is most likely because the envelopes of the exocytosed virions were damaged by the antiviral component present in the breast milk samples.

The apical surface of the infant’s tonsil epithelium is the first target for breast milk-mediated HIV-1 MTCT. Our findings, including the substantial reduction of HIV-1 binding to the apical surface of tonsil epithelium and complete inhibition of virus entry and transcytosis mediated by breast milk samples with antiviral effects, underscore its potential critical role in the inhibition of viral MTCT at its earliest stages. However, the significant increase in HIV-1 entry, transcytosis, and exocytosis in tonsil epithelium mediated samples that are devoid of antiviral effects suggests that breast milk may also promote HIV-1 MTCT.

The role of breast milk/calcium-induced PKC activation in increase of HIV-1 transcytosis/exocytosis in polarized tonsil epithelium suggests that calcium in breast milk may play a key role in promoting virus transmigration through the infant’s tonsil epithelium. This notion is supported by our current understanding of the direct contributions of the calcium sensor, SYT7, to viral transcytosis/exocytosis in polarized tonsil epithelial cells. SYT7 is extremely sensitive to calcium concentrations and plays a key role in the induction of exocytosis by facilitating vesicle fusion with the plasma membrane (MacDougall et al., 2018).

SYTs are transmembrane proteins with N-terminal transmembrane domains that anchor them to lipid bilayers (Shin et al., 2003; Sugita et al., 1996, 2001, 2002; Sugita and Sudhof, 2000). Elevated levels of cytoplasmic calcium result in a rapid increase in calcium binding to cytoplasmic domains C3A and C3B of the SYTs (Bhalla et al., 2005; Wang et al., 2005, 2006), leading to interactions with members of core membrane fusion machinery, including the N-ethyl-maleimide-sensitive factor attachment receptor proteins, syntaxin, and SPAP23/25 (Bhalla et al., 2006; Weber et al., 2014; Zhou et al., 2017). Calcium-bound SYTs also interact with anionic phospholipids on the plasma membranes that open fusion pores, thereby stimulating membrane fusion (Bhalla et al., 2005; Shin et al., 2003; Sugita et al., 1996, 2001, 2002; Sugita and Sudhof, 2000). Breast milk/calcium-induced colocalization of SYT7 and HIV-1 in tonsil cells suggests that SYTs might induce the formation of new HIV-containing vesicles (i.e., by fusion of SYT7-containing and HIV-containing vesicles). Breast milk/calcium-induced docking of HIV/SYT7 vesicles at plasma membranes revealed that activated SYT7 might facilitate docking of virus-containing vesicles and trigger their fusion with membranes during exocytosis.

In tonsil and gut tissue explants, breast milk samples lacking antiviral effects substantially increased the level of HIV-1 infection of subepithelial target cells, thereby confirming the critical role of breast milk/calcium/PKC-accelerated virus transmigration via mucosal epithelium, a cascade that may ultimately initiate the HIV-1 MTCT. Significant reductions in HIV-1 infection of subepithelial target cells mediated by breast milk samples with antiviral effect suggest that identification and further study of potential antiviral molecule(s) from these samples may uncover new strategies for the prevention of HIV MTCT.

**Fig. 10.**
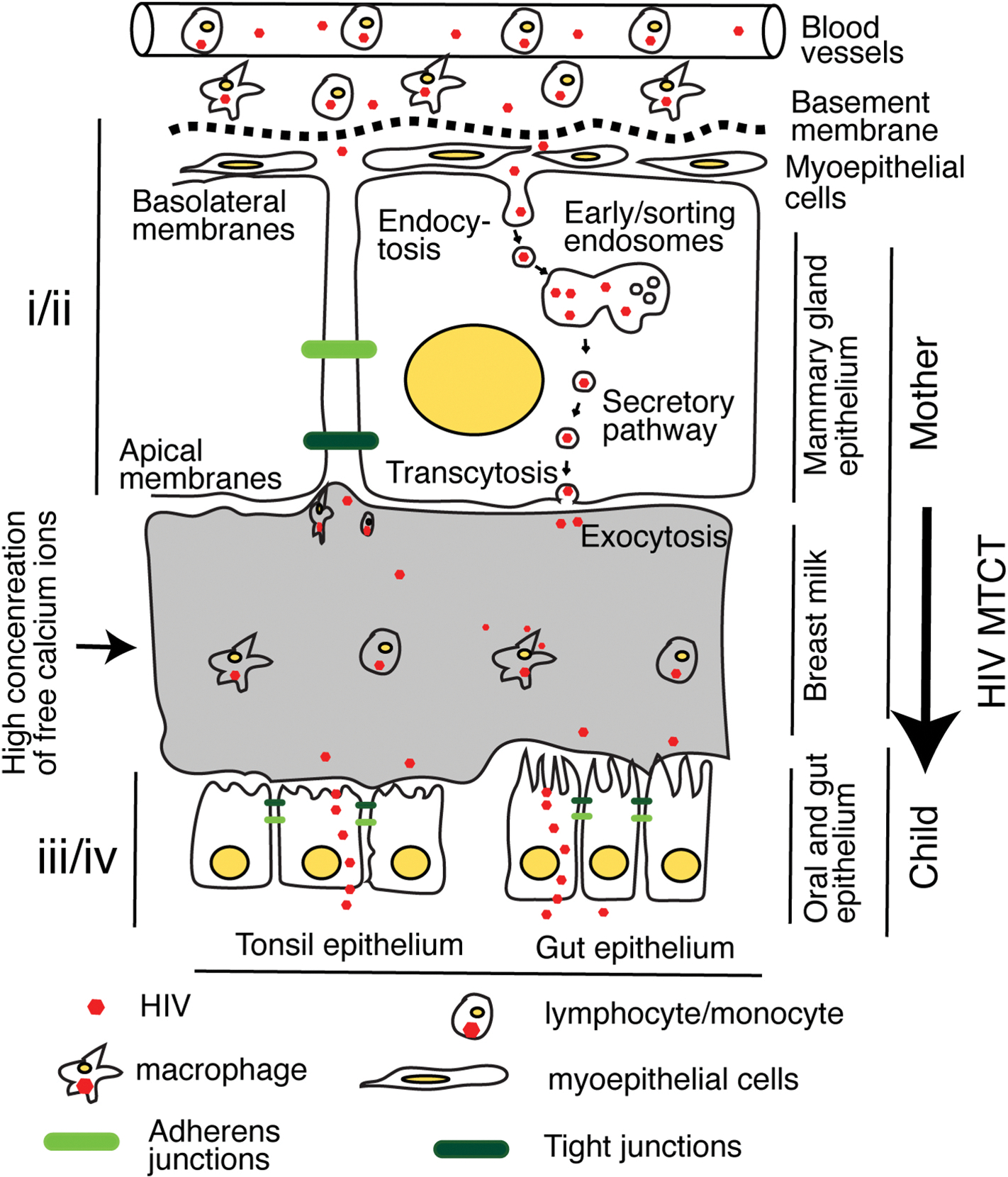
Model of breast milk-mediated HIV MTCT based on virus transfer via maternal MECs and infant tonsil/intestinal epithelial cells. MECs have a polarized organization and serve as a critical part of the blood-milk barrier during lactation. The apical membranes of MECs face the lumen, and the basolateral membranes are connected to underlying myoepithelial cells and blood vessels. Our findings suggest that HIV MTCT involves an interconnected multistep process that proceeds as follows: (i) cell-free HIV is transferred from the maternal circulation into breast milk via transcytosis through polarized MECs; and (ii) elevated concentrations of calcium ions or more factors in breast milk promote HIV exocytosis from MECs, as well as (iii) and (iv) HIV internalization, transcytosis, and exocytosis though infant tonsil and intestinal epithelial cells. High concentrations of calcium ions in breast milk facilitate HIV endocytosis in infant tonsils and gut epithelia. In response to calcium influx, SYT7 triggers vesicle-plasma membrane fusion, leading to the exocytosis of HIV-1 from tonsil/gut mucosal epithelial cells. Exocytosed virions can then infect submucosal CD4^+^ T lymphocytes and macrophages, substantially increasing MTCT of HIV.

Collectively, these findings suggest that breast milk samples collected from most lactating HIV-negative donors inactivate HIV and prevent virus infection of susceptible cells *in vitro* and in *ex vivo* tonsil and gut tissue explants. However, breast milk samples from nearly one quarter of the donors did not inactivate the HIV-1 but instead promoted viral transmigration through infant tonsils and fetal intestinal epithelial cells, a process that ultimately leads to infection of virus-susceptible subepithelial cells (Table 1, Fig. 10). Elevated concentrations of calcium in breast milk devoid of anti-HIV factors may play a critical role in promoting HIV-1 endocytosis, transcytosis, and exocytosis within the tonsil and gut mucosal epithelium, also leading to HIV-1 infection of virus-susceptible subepithelial cells. Nevertheless, we cannot yet rule out contributions from other possible proteins/factors contained in breast milk, some of which may also promote viral transmigration via infant tonsil/gut epithelia. These findings emphasize the need for further investigation focused on the identification of pro- and antiviral molecules in breast milk. These studies may expand our knowledge and provide a deeper understanding of the molecular pathogenesis and new strategies to prevent HIV MTCT.

## 6. Conclusions

Transfer of cell-free HIV-1 from the maternal circulation into breast milk is mediated by its basolateral-to-apical transcytosis of virus through polarized mammary epithelial cells (MECs) that function as a blood-milk barrier. While breast milk samples from 76 % of the HIV-negative donors contained factors that inactivated the virus by disrupting viral envelope glycoprotein gp120, thereby preventing MTCT, the remaining breast milk samples that were incapable of inactivating HIV-1 nonetheless induced viral exocytosis from the apical surface of MECs. Breast milk samples lacking anti-HIV activity promoted a substantial increase in viral internalization, transcytosis, and exocytosis from infant tonsils and gut epithelial cells, ultimately leading to infection of virus-susceptible subepithelial cells. Elevated calcium concentrations in breast milk play an important role in promoting HIV-1 entry, transcytosis, and exocytosis from infant tonsils and gut epithelial cells via activation of protein kinase C and calcium sensor synaptotagmin 7. Altogether, these findings revealed that breast milk lacking anti-HIV activity may promote HIV MTCT.

## Figures and Tables

**Table 1:** Summary of goals, models, experimental design, and outcomes.

Goals	Cells/tissue models, breast milk donors	Methods	Outcomes

To study the role of breast milk in HIV infectivity in breast milk cells	Breast milk white blood cells, CD4^+^ T lymphocytes, and CD14^+^ macrophages isolated from HIV-negative mothers. Breast milk samples and breast milk cells from donors BM#1 through BM#17.	HIV infectivity assay using dual tropic HIV-1_SF33_, X4 tropic HIV-1_92UG029_, and R5 tropic HIV-1_SF170_ viral strains	Breast milk from 76 % of donors completely or partially inhibited HIV-1 infection of breast milk cells. In contrast, breast milk from 24 % of donors exhibited no direct antiviral activity.
To study the mechanisms of HIV transmigration via the mother’s mammary epithelial cells	Polarized mammary epithelial cells.	HIV-1 endocytosis and transcytosis assays in mammary epithelial cells	Transmission of cell-free HIV-1 from the maternal circulation into breast milk is mediated by basolateral-to- apical transcytosis of the virus through polarized mammary epithelial cells.
To study the role of breast milk in HIV exocytosis of the mother’s mammary epithelial cells	Polarized mammary epithelial cells. Breast milk samples from donors BM#2, BM#4, BM#15, and BM#17.	HIV-1 exocytosis assays from the apical surfaces of mammary epithelial cells	Breast milk lacking anti-HIV effects facilitates HIV exocytosis from apical surfaces of mammary epithelial cells by activating calcium signaling.
To study the mechanisms of HIV endocytosis, transcytosis, and exocytosis via the infant’s tonsil epithelial cells	Polarized infant tonsil epithelial cells. Breast milk samples from donors BM#2, BM#4, BM#6, BM#13, BM#15, and BM#17.	HIV-1 attachment, endocytosis, transcytosis, and exocytosis assays	Breast milk lacking anti-HIV function substantially increases HIV endocytosis, transcytosis, and exocytosis in infant tonsil epithelia by activating calcium signaling and the calcium sensor synaptotagmin 7.
To study the mechanisms of HIV transmission via tonsil and intestinal mucosal epithelial tissues	Polarized oriented infant tonsil and fetal intestinal epithelial tissue explants. Breast milk samples from donors BM#2, BM#6, BM#15, and BM#17.	HIV transmigration and infection assays	Breast milk promotes HIV transmission through infant tonsil and gut epithelia, and leads to infection of virus- susceptible subepithelial cells, which is critical for HIV MTCT.

## Data Availability

All data are fully available without restriction.

## Data availability

Data will be made available on request.
